# Phosphorylation of CFP10 modulates *Mycobacterium tuberculosis* virulence

**DOI:** 10.1128/mbio.01232-23

**Published:** 2023-10-04

**Authors:** Basanti Malakar, Komal Chauhan, Priyadarshini Sanyal, Saba Naz, Haroon Kalam, R. P. Vivek-Ananth, Lakshya Veer Singh, Areejit Samal, Dhiraj Kumar, Vinay Kumar Nandicoori

**Affiliations:** 1 National Institute of Immunology, Aruna Asaf Ali Marg, New Delhi, India; 2 International Centre for Genetic Engineering and Biotechnology, Aruna Asaf Ali Marg, New Delhi, India; 3 Academy of Scientific and Innovative Research (AcSIR), CSIR-Centre for Cellular and Molecular Biology Campus, Hyderabad, India; 4 The Institute of Mathematical Sciences (IMSc), Homi Bhabha National Institute (HBNI), Chennai, India; Max Planck Institute for Infection Biology, Berlin, Germany

**Keywords:** tuberculosis, secretion, phosphorylation, virulence, CFP10, ESAT6, PknA, PknB

## Abstract

Virulence effectors secreted by *Mycobacterium tuberculosis* (*Mtb*) help subvert host immune mechanisms and, therefore, are critical for establishing infection and pathogenesis. However, knowledge in terms of signaling mechanisms that modulate the secretion of virulence factors is sparse. We performed high-throughput secretome, phosphoproteome, and phospho-secretome analysis of *Mtb*. We combined the analysis with empirical validations to show regulation of mycobacterial secretion through protein phosphorylation. System level protein-protein interaction network analysis superimposed with the secretome, phosphoproteome, and phospho-secretome profile revealed an intricate relationship between phosphorylation and secretion. At the core of the network was a key virulence factor CFP10. We identified PknA to be the kinase responsible for phosphorylating CFP10. Using genetic tools, we show that phosphomimetic mutation of CFP10 negatively regulates the secretion of virulence mediator ESAT6. Significantly, the dynamics of CFP10 phosphorylation strongly influenced bacterial virulence and survival within macrophages and mice. Together, the results show that the dynamic phosphorylation status of the secretory protein CFP10 regulates the secretion of virulence factors and impacts virulence.

**IMPORTANCE:**

Secreted virulence factors play a critical role in bacterial pathogenesis. Virulence effectors not only help bacteria to overcome the host immune system but also aid in establishing infection. *Mtb*, which causes tuberculosis in humans, encodes various virulence effectors. Triggers that modulate the secretion of virulence effectors in *Mtb* are yet to be fully understood. To gain mechanistic insight into the secretion of virulence effectors, we performed high-throughput proteomic studies. With the help of system-level protein-protein interaction network analysis and empirical validations, we unravelled a link between phosphorylation and secretion. Taking the example of the well-known virulence factor of CFP10, we show that the dynamics of CFP10 phosphorylation strongly influenced bacterial virulence and survival *ex vivo* and *in vivo*. This study presents the role of phosphorylation in modulating the secretion of virulence factors.

## INTRODUCTION

*Mycobacterium tuberculosis* (*Mtb*) possesses the ability to modify host defense mechanisms at multiple stages of infection, often through its vast repertoire of secretory molecules. Due to the presence of an unusual outer membrane, the bacterium has evolved different secretion systems to deliver effectors into the host. Like most bacteria, *Mtb* harbors a general secretory system (Sec) to majorly export unfolded proteins by recognizing an N-terminal signal sequence. Mycobacteria also harbor two paralogs of the translocase SecA, SecA1, and SecA2 (1); SecA2 is prevalent in Gram-positive pathogenic bacteria (2). Proteins secreted through SecA1 system include RipA (a peptidoglycan-hydrolyzing endopeptidase), LprG, LpqH (lipoproteins), and other virulence factors with unknown function (3 – 9). SecA2 in mycobacteria is reported to export Mce transporters, antioxidants like SodA and KatG, and many other substrates, including SapM and PknG (10 – 12). Recently, SatS was shown to act as a protein export chaperone and helps export SapM and other substrates of SecA2 pathway (13). SecA2 plays an essential role in arresting phagosome maturation and promoting bacterial growth inside macrophages (14 – 16). The other secretory system, which primarily transports folded proteins across the inner membrane, is the twin-arginine pathway or Tat pathway, named because of the twin-arginine motif in the signal sequence (17). Four phospholipase C enzymes and Rv2525c, a conserved hypothetical protein, are known Tat substrates (17, 18) in mycobacteria. In addition to the classical Sec and Tat secretion system, most Gram-negative bacteria are equipped with a unique system to deliver their contents to the host. Such a specialized secretion apparatus ranges from the type I-VI system (19). Most of the mycobacterial species, firmicutes such as *Staphylococcus aureus*, *Streptomyces coelicolor*, *Bacillus subtilis*, utilize a specialized secretory system named Type VII secretion system (T7SS, also known as Esx systems) (20 – 22).

*Mtb* possesses five Esx systems, namely, Esx-1–5, out of which three are essential for virulence (23 – 26). Mycobacterial Esx systems are associated with pathogenicity in many species, of which *esx-1* locus is the most extensively studied because of its role in virulence and host-pathogen interaction (27 – 30). CFP10 (Rv3874, also known as EsxB) and ESAT6 (Rv3875, also known as EsxA) are the major substrates of Esx-1 machinery that interact with each other (31). They are also commonly known as the WXG-100 family of proteins because of a tryptophan-X-glycine motif (32). A conserved YxxxD/E motif in the C-terminus of CFP10 is essential for interaction with EccC ATPase, which transports the complex toward the membrane (32, 33). With the help of experiments using recombinant ESAT6, the protein was shown to possess cytolytic activity inside the host cell (28, 34, 35). While ESAT6 alone is not sufficient for membrane disruptions, its presence is necessary along with other Esx-1 effectors to carry out contact-dependent lysis of host cell membrane (36). CFP10 by itself was shown to induce chemotaxis of neutrophils through activation of the G-protein coupled receptor (37). CFP10 or ESAT6 or both together downregulate NF-κB signaling through inhibition of ROS production (38). Furthermore, the CFP10-ESAT6 complex also downregulates autophagy, a host counteractive mechanism to contain intracellular pathogen (39, 40).

Reversible protein phosphorylation controls biological processes ranging from metabolism and cellular homeostasis to cell wall biosynthesis and division (41). *Mtb* has an elaborate arsenal of phosphosignaling molecules, including 12 two-component systems and 11 eukaryotic-like serine threonine protein kinases (STPKs) along with 1 serine-threonine phosphatase, PstP; 1 tyrosine kinase, PtkA; and 2 tyrosine phosphatases, PtpA and PtpB (42, 43). Studies have suggested the importance of transcriptional regulators, like EspR, in secretion of T7SS substrates, through activation of the accessory operon *espACD* (44). EspJ, a T7SS protein, is hyper-phosphorylated in the pathogenic strain, *H37Rv*, and phosphorylation influences virulence (45). With the help of high-resolution mass spectrometry, Parra et al. identified an unprocessed phosphorylated form of LpqH, a secretory lipoglycoprotein (46). However, the regulatory mechanisms delineating the functional implications of secretory protein phosphorylation are relatively unexplored.

Here, we sought to decipher the association between phosphorylation and secretion. With the help of a high-throughput proteomic approach, we identified the global phosphoproteome and secretome of *Mtb*. We utilized the protein-protein interaction (PPI) network to capture the interactions between phosphorylated and secreted proteins. We report here that CFP10, one of the most well-characterized and essential factors for the pathogen’s virulence, gets phosphorylated, which, in turn, impacts secretion of ESAT6 and, thereby, results in differential bacterial virulence and survival in macrophages and animal models.

## RESULTS

### Phosphoproteome of Mtb

**Fig 1:**
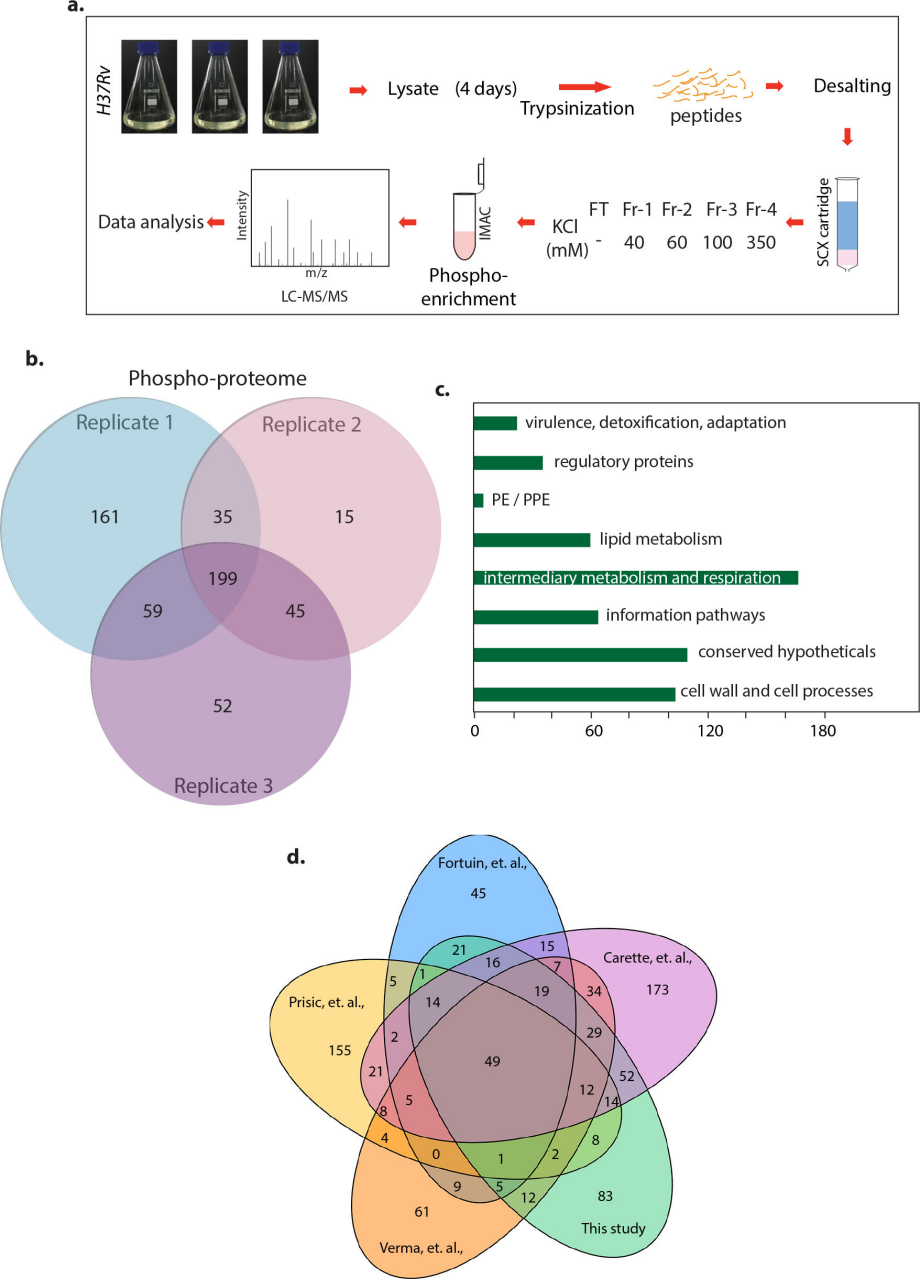
Phosphoproteome of Mtb. (a) Schematic workflow of the phosphoproteomic experiment. (b) Venn diagram showing phosphorylated proteins in all three biological replicates. (c) Functional classification of *Mtb* phosphoproteins according to the Mycobrowser database. *X* axis denotes number of proteins identified in particular categories. (d) Venn diagram showing the size of the phosphoproteome of the current study and four other published studies. Turapov et al. study was not included in the comparative analysis due to lack of Uniprot IDs (50).

We set out to delineate the role of phosphorylation in modulating the function of secretory proteins. We performed phosphoproteome and secretome analysis of *Mtb H37Rv* grown in nutrient limiting Sauton’s media. The samples were trypsinized, fractionated, enriched for phosphopeptides and analyzed by mass spectrometry (Fig. 1a). The experiment performed in biological triplicates led to the identification of 903 phosphosites corresponding to 566 unique *Mtb* proteins (Fig. 1b; Data Sets S1 and S2). Among them, 338 proteins were detected in any 2 biological replicates, and 199 were found in all 3 biological triplicates (Fig. 1b). The phosphorylated proteins could be classified into eight out of the nine major functional categories according to Mycobrowser database, with intermediary metabolism being a major class (Fig. 1c). Unlike previous studies wherein threonine phosphorylation was found to be the primary target (47 – 51), we found serine to be the major target in our study (serine:threonine:tyrosine-52.4:45.4:2.2; Data Set S2). In addition to this study, five different studies have reported high-throughput phosphoproteomics of *Mtb H37Rv* under different culture conditions (47 – 51). We found 49 proteins to be common in 5 studies, including this study, and 83 unique proteins were identified for the first time in our study (Fig. 1d; Data Set S3a).

### Secretome of Mtb

**Fig 2:**
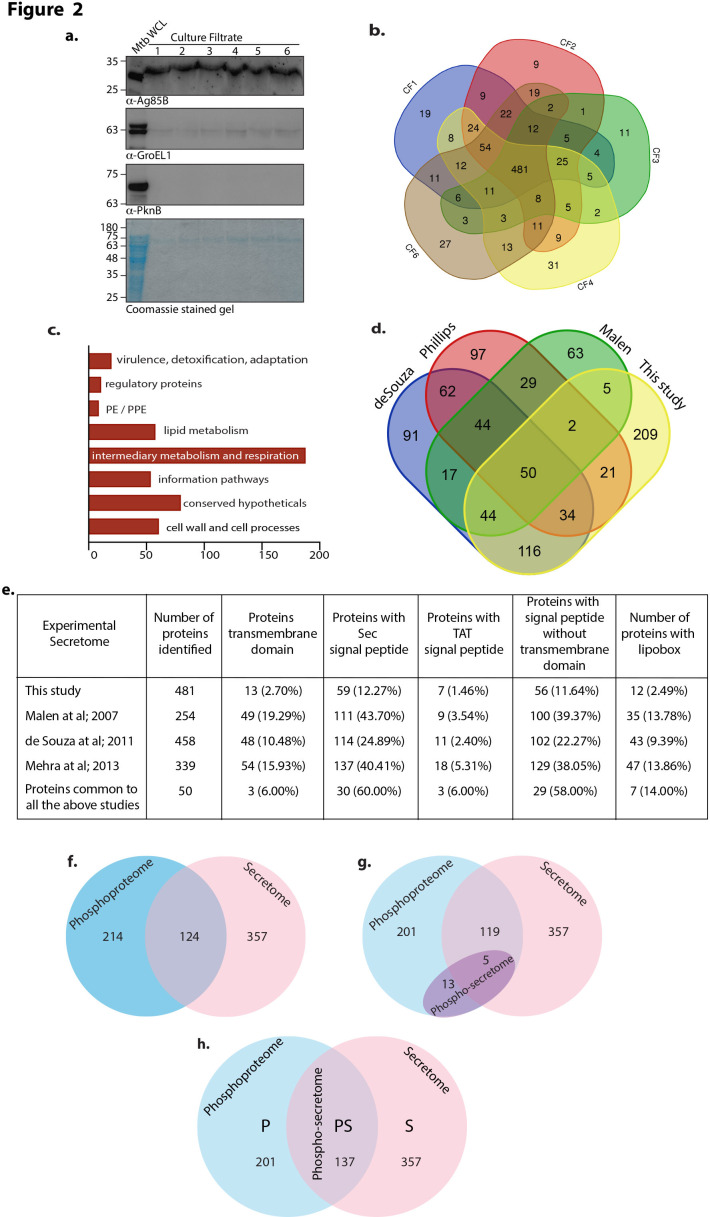
Secretome of Mtb. (a) Twenty micrograms of lysate and culture filtrates from logarithmic phase *H37Rv* culture was resolved, transferred to nitrocellulose membrane, and probed with α-Ag85B, α-GroEL1, and α-PknB antibodies. (b) Venn diagram showing the secreted proteins in all five biological replicates. (c) Functional classification of *Mtb* secretory proteins according to the Mycobrowser database. *X* axis denotes number of proteins identified in particular categories. (d) Venn diagram showing the size of the secretome of the current study and three other published studies. Putim et al. study was not included in the comparative analysis due to lack of Uniprot IDs (55). (e) Number of secreted proteins in each experimental study with predicted Sec signal peptide, TAT signal, lipobox domain, signal peptide prediction, with transmembrane prediction and with signal peptide prediction but no transmembrane prediction. (f) Venn diagram showing the overlap of proteins between phosphoproteome and secretome lists. (g) Venn diagram showing the overlap of newly introduced phospho-secretome data into the pre-existing phosphoproteome and secretome lists. (h) Venn diagram showing the final number of proteins that were phosphorylated, secreted, or both phosphorylated and secreted.

We verified that the culture filtrates were free of cytosolic proteins by probing for Ag85B (secreted) and PknB (non-secreted) in the lysate and culture filtrate samples (Fig. 2a). While most of the GroEL1 was in the cytosol, traces of GroEL1 were also found in the culture filtrate, which was consistent with other reports (52). We evaluated the total secretome of *Mtb H37Rv* by harvesting culture supernatants and analyzing tryptic digests by mass spectrometry. Overall, we used 5 biological replicates to identify a total of 862 proteins, with 481 proteins being common to all 5 independent biological replicates (Fig. 2b; Data Set S4). The secretory proteins were distributed as per Mycobrowser functional categories, wherein we did not find proteins belonging to insertion sequence and phages (Fig. 2c). The major functional classes enriched in the secretome list were intermediary metabolism and cell wall and cell processes (Fig. 2c). We compared the secretome profile with those from three previously published *Mtb H37Rv* secretome studies (52 – 54) and found that 50 proteins were common to all 4 studies (Fig. 2d). Among the 481 secretory proteins identified in this study, 272 proteins have been reported in any 1 or more of the previous studies (Fig. 2d). We also identified 209 unique proteins that have not been identified in any of the previous studies (Fig. 2d; Data Set S5).

Secreted proteins reported by each study were annotated with bioinformatics-based predictions on secretion signals such as a signal peptide, TAT signal, and Lipobox domain (see Materials and Methods) (Fig. 2e). We also assessed the number of proteins with a putative signal peptide containing the transmembrane domain and a putative signal peptide without a transmembrane domain (see Materials and Methods) (Fig. 2e). Analysis of abundance of antigenic regions (AAR) suggested that secreted proteins in *Mtb* are likely to be more antigenic than the entire proteome (see Materials and Methods) (Fig. S1). Taken together, in the nutrient limiting media, we identified a total of 566 phosphoproteins and 862 secretory proteins. However, we considered 338 phosphoproteins and 481 secretory proteins to ensure consistency for subsequent analysis.

### Phosphoproteome and secretome

Analysis of the phosphoproteome and secretome data showed that 124 proteins are part of both the lists (Fig. 2f; Data Set S6). This observation opened up an exciting facet, a possible relationship between phosphorylation and secretion. To empirically explore the connection between phosphorylation and secretion, we performed phospho-secretome analysis by enriching phosphopeptides in the *Mtb H37Rv* culture filtrate tryptic digests and identified 33 phosphosites in 18 proteins (Data Sets S7 and S8). In contrast to results from the lysates (Data Set S2), we found the distribution of serine:threonine:tyrosine to be 32:57:11, with threonine being the primary target. The 18 phosphoproteins of the secretome were a subset of 338 phosphoproteins from the lysates (Fig. 2g). Interestingly, 5 of these proteins, namely, Rv1827, Rv2204c, Rv2711, Rv3874, and Rv3875, were part of 290 secretory proteins (Fig. 2g; Data Sets S7 and S8), affirming that these proteins are, indeed, phosphorylated and secreted. The phospho-secretome analysis identified 13 additional phosphorylated proteins (Rv0007, Rv0020c, Rv0206c, Rv0461, Rv0810c, Rv1638A, Rv1747, Rv2151c, Rv2536, Rv2921c, Rv3814c, Rv2198c, and Rv3910). These proteins increased secretome and phosphorylated secreted proteins to 494 and 137, respectively (Fig. 2h). Among the 338 phosphorylated proteins, 201 were phosphorylated but not part of the secretome. In 494 secretome proteins, 357 were part of the secretome but not the phosphoproteome. One hundred thirty-seven proteins were present in both phosphoproteome and secretome (Fig. 2h).

### Protein-protein interaction network analysis reveals an intricate relationship between phosphorylation and secretion

**Fig 3:**
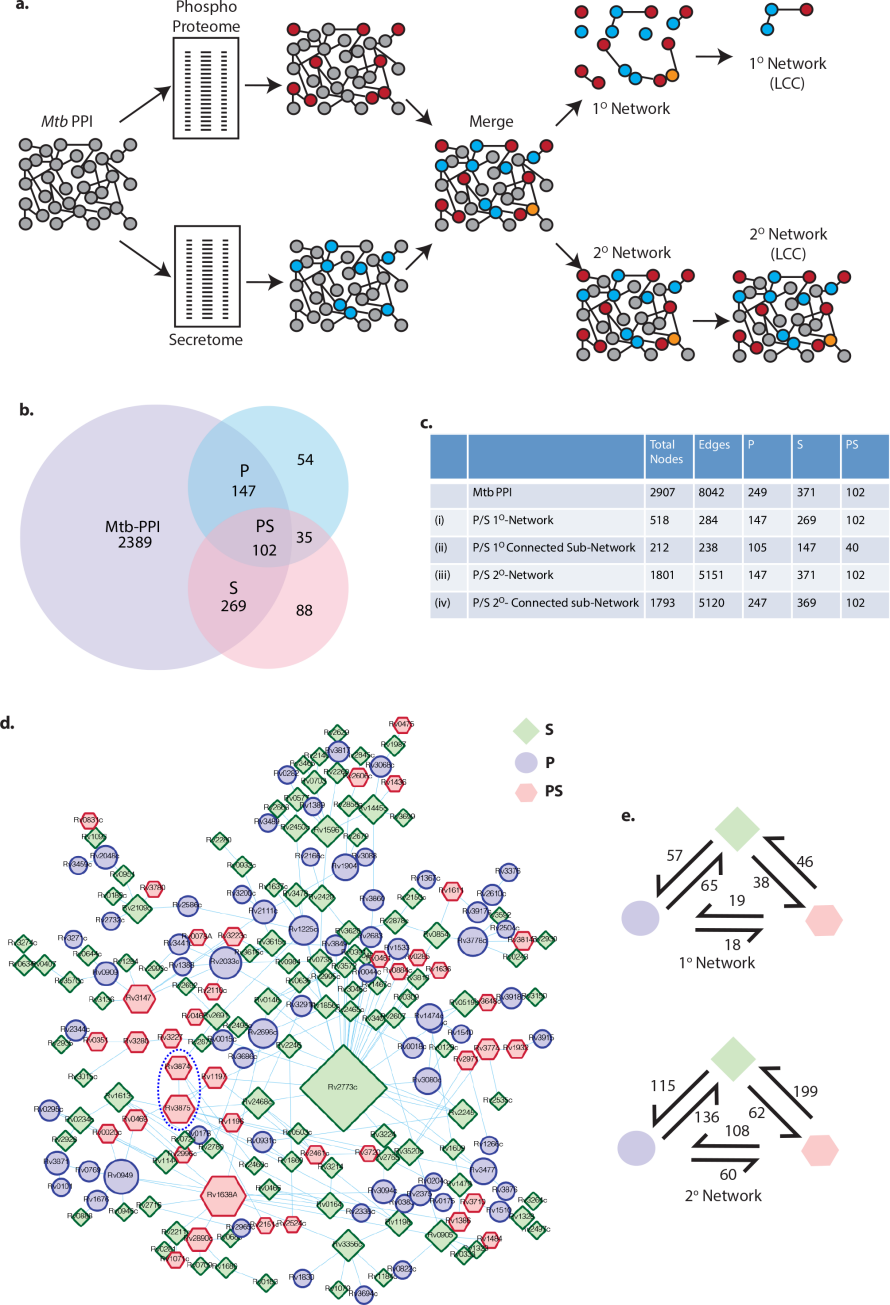
Protein-protein interaction network analysis reveals an intricate relationship between phosphorylation and secretion. (a) Proteins that were present in the phosphoproteome, secretome, or phospho-secretome lists were incorporated in the *Mtb* PPI network from Wang et al. (57) using Cytoscape 3.2.0. The iterative steps followed to enrich the proteins from the experimental data into the network, and further filtering of interacting nodes is schematically shown in the flowchart (red, phosphor-proteome “P”; blue, secretome “S”; orange, common to both phosphoproteome and secretome “PS”). (b) Venn diagram showing the overlap of phosphorylated (P), secreted (S), and phosphorylated and secreted (PS) proteins with the PPI network. (c) Basic network properties of each of the one- and two-degree networks obtained through the exercise shown in 2a. The number of P, S, and PS proteins present in each of these networks is shown in c. (d) The largest connected sub-network in the one-degree network. Diamonds, circles, and hexagons denote P, S, and PS proteins, respectively. The size of nodes corresponds to their stress centrality value in the parent network (larger the stress coefficient, bigger the size). (e) Nodes appearing in each of the extracted sub-networks in Fig. 2c were analyzed in terms of connectivity among P, S, and PS proteins, and the corresponding counts of such interactions are shown in 2e.

Next, we explored the association between phosphoproteome and secretome using *Mtb* protein-protein interaction network. The 201 phosphorylated, 357 secreted, and 137 phospho-secreted proteins were mapped onto the PPI network (Fig. 3a) (56) . The iterative steps performed to enrich the proteins from the experimental data into the network and further filtering of interacting nodes are depicted in the flowchart (Fig. 3a). This network contains 2,907 *Mtb* proteins comprising 8,042 interactions. In the PPI network, we could map 147 phosphorylated (P) proteins (out of 201), 269 secreted (S) proteins (out of 357), and 102 proteins (out of 137) that are both phosphorylated and secreted (PS) (Fig. 3b; Data Set S6).

The phosphoproteome-secretome-phospho-secretome network allowed us to perform some fundamental graph-theoretic analysis to unravel any underlying relationship between these sets of proteins. The details of the graph-theoretic analysis strategy (Fig. 3a) are as follows. All the proteins and interactions that were present in either phosphorylated (P) or secreted (S) or phosphorylated and secreted (PS) data sets were retained (Fig. 3a). This network is defined as a one-degree network since no additional proteins other than those present in the three lists were retained. We next extracted the most significant connected component of the one-degree network to achieve what we call a one-degree connected sub-network (Fig. 3a). At the next iteration, we allowed one additional protein to be retained in the interaction network if that helped connect two proteins from our lists, resulting in a two-degree network (Fig. 3a). Similar to the first iteration, we subsequently extracted the largest connected component to get a two-degree connected sub-network (Fig. 3a). While a one-degree network had 147 P, 269 S, and 102 PS proteins, only 105 P, 147 S, and 40 PS proteins among them were retained in the connected one-degree network. On the other hand, all P, S, and PS proteins were retained in a two-degree connected sub-network, except for 2 P and 2 S proteins (Fig. 3c). The one-degree connected sub-network, thus, generated is depicted in Fig. 3d. The node size in this representation reflects their corresponding value for a critical centrality parameter, i.e., stress centrality, calculated for the entire *Mtb* network. A comparative analysis of connectivity between P, S, and PS proteins in one- and two-degree networks is depicted in Fig. 3e. The connectivity between P, S, and PS molecules increases substantially in the two-degree network compared with the one-degree network.

The simplistic inference from this intriguing interaction pattern between P, S, and PS proteins could be that the two processes of protein phosphorylation and protein secretion in *Mtb* are strongly linked. Among the PS proteins, Rv1638, Rv1197, Rv3874, and Rv3875 showed a high-stress coefficient and high node degree, i.e., interactions (Fig. 3d). Interestingly, Rv3874 and Rv3875 (Fig. 3d; indicated by dotted blue line), also known as CFP10 and ESAT6, are well-known virulence factors of *Mtb* secreted through the T7SS (37, 38, 58, 59). The phosphorylation of secretory proteins including CFP10 and ESAT6 has been reported previously (57), but, the correlation between phosphorylation, secretion, and implications to virulence has not been rigorously investigated. Furthermore, while ESAT6 was shown to be phosphorylated by PknB, the kinase responsible for phosphorylating CFP10 has not been identified (57).

### PknA majorly phosphorylates CFP10

**Fig 4:**
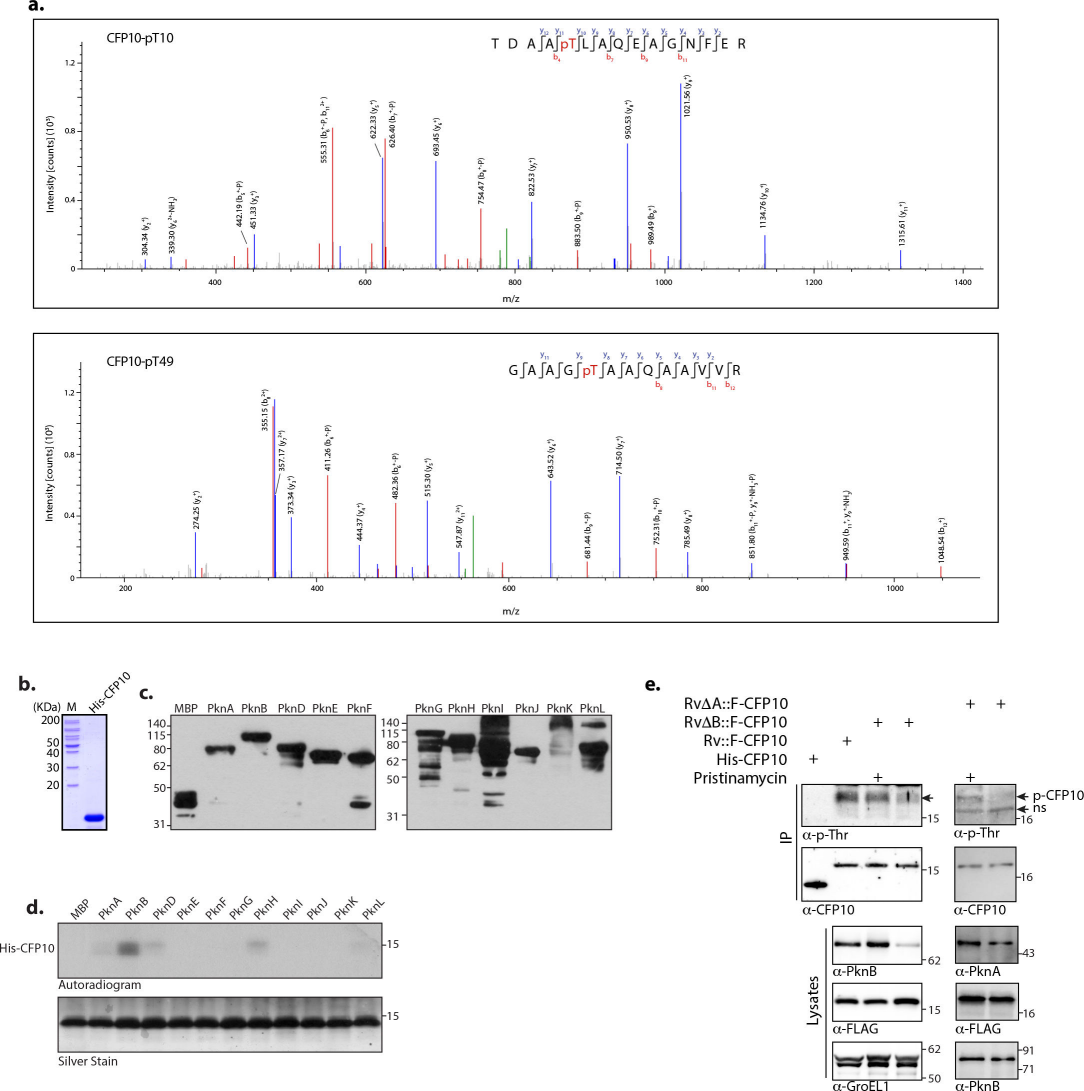
CFP10 is phosphorylated by PknA. (a) MS/MS spectrum of phosphopeptides corresponding to T10 and T49 of CFP10, respectively. MS/MS spectrum of precursor *m*/*z*: 837.36743 (+2) and MH+: 1673.72759 Da of the phosphopeptide TDAA(pT)LAQEAGNFER from protein CFP10. The location of the intact phosphate group on T10 was confirmed by the observation of “b” and “y” ion series containing b_4_, b_7_, b_9_, b_11_, and y_2–12_. MS/MS spectrum of precursor *m*/*z*: 611.80048 (+2) and MH+: 1222.59368 of the phosphorylated peptide GAAG(pT)AAQAAVVR from protein CFP10. The location of T49 was evident from the ion series containing b_8_, b_11_, b_12_, y_2–9_, and y_11_. (b) Coomassie stained gel showing purified His-CFP10. (c) *E. coli* BL21 cells were transformed with pDuet-kinase constructs, and WCLs were prepared after induction with 1 mM IPTG. Ten micrograms of crude extract was resolved, transferred to nitrocellulose membrane, and probed with α-MBP antibody. (d) *In vitro* kinase assays were performed with 1–2.5 μg WCLs depending on the expression level of kinase and 2 µg His-CFP10 (170 pmol) in the presence of 10 µCi [32P]ATP and 10 µM ATP. Samples were resolved on 6 M urea containing 16% SDS-PAGE, autoradiographed (top panel), and silver stained (bottom panel). (e) *Rv*, *Rv*Δ*A,* and *RvΔB* strains were electroporated with pNit-F-cfp10. WCLs were prepared from cultures initiated at *A*_600_ of 0.1 and grown in the presence or absence of pristinamycin (100 ng/mL) and anhydrotetracycline (ATc) (1.5 µg/mL). Five micromolars of isovaleronitrile (IVN) was added to the cultures to induce the expression of F-CFP10. 10, 10, 10, and 50 µg of WCLs were resolved on SDS-PAGE; transferred to nitrocellulose membrane; and probed with α-PknA, α-PknB, α-GroEL1, and α-FLAG antibodies, respectively. For immunoprecipitation (IP), 1 mg of each sample was immunoprecipitated using FLAG beads. One-tenth of the IP sample was probed with α-CFP10 antibody. Rest 9/10th sample was probed with α-p-Thr antibody. His-CFP10 was used as a negative control for phosphorylation. Phosphorylated CFP10 is indicated by an arrow.

In three biologically independent experiments, we found CFP10 to be phosphorylated on T10 and T49 residues. Liquid chromatography-mass spectrometry (LC-MS) analysis of CFP10 tryptic peptides showed precursor mass-to-charge ratio of 837.37 and 611.80, corresponding to the mass of doubly charged phosphopeptides from residues 6–20 and 45–57 aa, respectively (Fig. 4a). In addition to these sites, S84 residue in CFP10 is phosphorylated in *H37Rv* Beijing isolate (*Mtb* isolate, SAW5527) (48). After identifying the target phosphorylation sites in CFP10, we sought to identify the kinase responsible for phosphorylating it. We have previously generated pDuet constructs expressing either MBP-tagged full-length protein kinases (PknA, PknB, PknK, and PknG) or MBP-tagged protein kinases up to the intracellular transmembrane domain (PknD–F, PknH–J, and PknL) (59). CFP10 was purified as N-terminal hexa His-tagged protein (Fig. 4b), and *in vitro* kinase assays were performed with *Escherichia coli* lysates expressing MBP-tagged kinases. Western blot analysis was performed using α-MBP antibody to normalize the amount of lysate used for the kinase reaction (Fig. 4c). Results show that CFP10 is robustly phosphorylated by PknB and to an extent by PknA, PknD, and PknH (Fig. 4d).

Taking cues from the *in vitro* experiment, we set out to validate the findings using previously reported pristinamycin-inducible *pknA* (*Rv*Δ*A*) and *pknB* (*Rv*Δ*B*) conditional mutant strains in *H37Rv* (60, 61). When the cultures were grown in the absence of pristinamycin, these strains showed depletion of either PknA or PknB. We electroporated pNit-F-cfp10 construct into these strains; lysates were prepared from cultures grown for 3 days either in the presence or in the absence of the inducer. As anticipated, expression of PknA and PknB was significantly lower in the absence of inducer, as evident from the lysates western blot (Fig. 4e, bottom panel, compare lane 3 with 2 for PknB and lane 5 with 4 for PknA). To evaluate the expression and phosphorylation, FLAG-tagged CFP10 was immunoprecipitated and probed with α-CFP10 and α-p-Thr antibodies. We loaded purified His-CFP10 as a control along with immunoprecipitated FLAG-CFP10 (Fig. 4e, top two panels, first lanes). While the anti-CFP10 antibody is expected to detect both His- and FLAG-tagged CFP10, the p-Thr antibody should only detect immunoprecipitated FLAG-CFP10 from *Mtb* lysates as His-CFP10 purified from *E. coli* will not be phosphorylated (Fig. 4e, top two panels). Results show that the p-Thr antibody specifically detects phosphorylated p-F-CPF10 but not the His-CFP10. It is apparent from the data that depletion of either PknB (Fig. 4e, top two panels, compare lane 4 with 3) or PknA results in decreased intensity of F-CFP10 phosphorylation. However, the difference observed with PknA depletion was more pronounced (Fig. 4e, top two panels, compare lane 6 with 5). Results suggest that while both PknA and PknB are capable of phosphorylating CFP10, PknA is likely to the primary kinase involved in its phosphorylation.

### Phosphorylation of CFP10 does not affect its interaction with ESAT6

**Fig 5:**
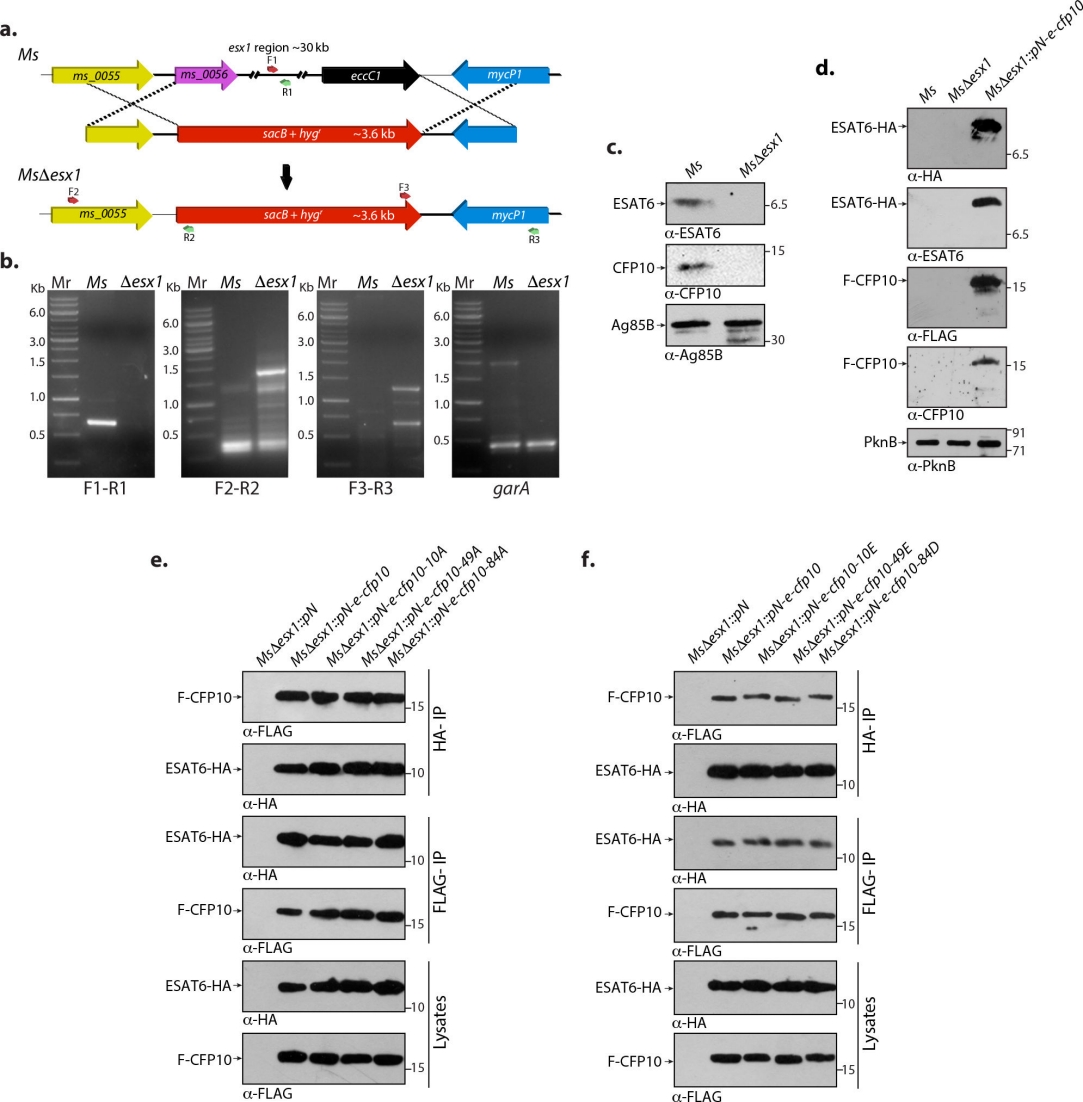
Phosphorylation of CFP10 does not affect its interaction with ESAT6. (a) Schematic depiction of the strategy used for the generation of gene replacement mutants. Primers used for PCR confirmation are indicated. (b) Genomic DNA was isolated from log-phase cultures of wild-type and mutant strains, and PCRs were carried out with indicated sets of primers. Mr denotes 1 kb ladder. First panel shows amplification of *cfp10-esat6* (0.6 kb) in *Ms* but not in *Ms*Δ*esx-1*. Second and third panels show differential PCR amplification (1.6 and 1.2 kb, respectively) obtained in *Ms*Δ*esx-1* but not in *Ms*. The last panel shows the control *garA* amplicon (0.4 kb) in both the strains. (c) *Ms* and *Ms*Δ*esx-1* strains were grown in Sauton’s media till the logarithmic phase, and CFPs were prepared. 50 µg (for CFP10 and ESAT6) and 20 µg (for Ag85B) of CFPs were resolved; transferred on nitrocellulose membrane; and probed with α-ESAT6, α-CFP10, and α-Ag85B antibodies. (d–f). *Ms*Δ*esx-1* strain was electroporated with pN-e-cfp10 or pN-e-cfp10-10A, or pN-e-cfp10-10E, pN-e-cfp10-49A, or pN-e-cfp10-49E or pN-e-cfp10-84A, or pN-e-cfp10-84D constructs expressing CFP10-ESAT6 or CFP10_mutants_-ESAT6 in episomal pNit-3F (IVN inducible) vector to generate *Ms*Δ*esx-1::pN, Ms*Δ*esx-1::pN-e-cfp10, Ms*Δ*esx-1::pN-e-cfp10-10A, Ms*Δ*esx-1::pN-e-cfp10-10E, Ms*Δ*esx-1::pN-e-cfp10-49A, Ms*Δ*esx-1::pN-e-cfp10-49E, Ms*Δ*esx-1::pN-e-cfp10-84A,* and *Ms*Δ*esx-1::pN-e-cfp10-84D* strains. (d) Fresh cultures of *Ms*, *Ms*Δ*esx-1,* and *Ms*Δ*esx-1::pN-e-cfp10* were seeded at an initial *A*_600_ of 0.1 and induced with 5 µM IVN for 10 h. WCLs were resolved and probed with α-HA, α-ESAT6, α-FLAG, α-CFP10, and α-PknB antibodies. (e) Twenty micrograms of WCLs was prepared from *Ms*, *Ms*Δ*esx-1::pN, Ms*Δ*esx-1::pN-e-cfp10, Ms*Δ*esx-1::pN-e-cfp10-10A, Ms*Δ*esx-1::pN-e-cfp10-49A,* and *Ms*Δ*esx-1::pN-e-cfp10-84A* grown in the presence of 5 µM IVN; resolved; and probed with α-HA and α-FLAG antibodies (bottom panel). One milligram protein of each sample was immunoprecipitated using FLAG beads. One-tenth of the IP sample was probed with α-HA and α-FLAG antibodies (middle panel). One milligram of each WCLs was immunoprecipitated using HA beads, and 1/10th of the IP sample was probed with α-FLAG and α-HA antibodies (top panel). (f) Twenty micrograms of WCLs was prepared from *Ms*, *Ms*Δ*esx-1::pN, Ms*Δ*esx-1::pN-e-cfp10, Ms*Δ*esx-1::pN-e-cfp10-10E, Ms*Δ*esx-1::pN-e-cfp10-49E,* and *Ms*Δ*esx-1::pN-e-cfp10-84D* grown in the presence of 5 µM IVN, resolved, and probed with α-HA and α-FLAG antibodies (bottom panel). One milligram protein of each sample was immunoprecipitated using FLAG beads. One-tenth of the IP sample was probed with α-HA and α-FLAG antibodies (middle panel). One milligram of each WCLs was immunoprecipitated using HA beads, and 1/10th IP sample was probed with α-FLAG and α-HA antibodies (top panel). For e and f panels, representative data of two independent experiments are presented.

Next, we sought to validate if protein phosphorylation could impact secretion in mycobacteria. CFP10-ESAT6 forms a four-helix bundle due to their individual helix-turn-helix structure to form a heterodimeric complex inside cytoplasm (31). In order to evaluate the role of phosphorylation in modulating CFP10-ESAT6 interaction, it is essential to retain the proteins in the cytosol. To achieve this objective, we generated an *M. smegmatis* Δ*esx-1* (*Ms*Δ*esx-1*) mutant by deleting the 30 kb region between *ms_0056* and *ms_0082* by allelic exchange (Fig. 5a) (62). The fidelity of homologous recombination at the native locus was confirmed by performing PCR reactions using appropriate sets of primers (Fig. 5b). Western blot analysis of culture filtrate proteins (CFPs) from *Ms* and *Ms*Δ*esx-1* strains with α-CFP10, α-ESAT6, and α-Ag85B (control) confirmed the deletion (Fig. 5c). *cfp10-esat6* operon was cloned into the pNit-3F, such that CFP10 and ESAT6 would be expressed with an N-terminal 3×-FLAG-tag and a C-terminal 3×-HA-tag, respectively. Western blot analysis showed efficient expression and retention of F-CFP10 and ESAT6-HA in the cytosol of *Ms*Δ*esx-1::pN-e-cfp10* (Fig. 5d). Next, we generated phosphoablative (T or S→A) and phosphomimetic (T or S→E or D, respectively) mutants of CFP10T10, CFP10T49, and CFP10S84 and cloned them into pNit-3F vector. The constructs were transformed into the *Ms*Δ*esx-1* strain, lysates were prepared, and F-CFP10 or ESAT6-HA were immunoprecipitated (IP) with anti-FLAG-M2 or anti-HA agarose beads, respectively. We observed efficient expression of wild-type and phosphoablative and phosphomimetic mutants of CFP10 (Fig. 5e and f; bottom panels). We evaluated the interaction by probing the FLAG-IP with α-HA and HA-IP with α-FLAG antibodies. It is apparent that mutating T10, T49, and S84 residues of CFP10 to alanine residues did not alter their interaction (Fig. 5e). Mutating T10, T49, and S84 residues of CFP10 to phosphomimetic E or D residues also did not influence its interaction with ESAT6 (Fig. 5f). Collectively, the data suggest that phosphorylation of CFP10 at T10, T49, or S84 does not influence its binding with ESAT6.

### Phosphorylation of CFP10 negatively modulates secretion of ESAT6

**Fig 6:**
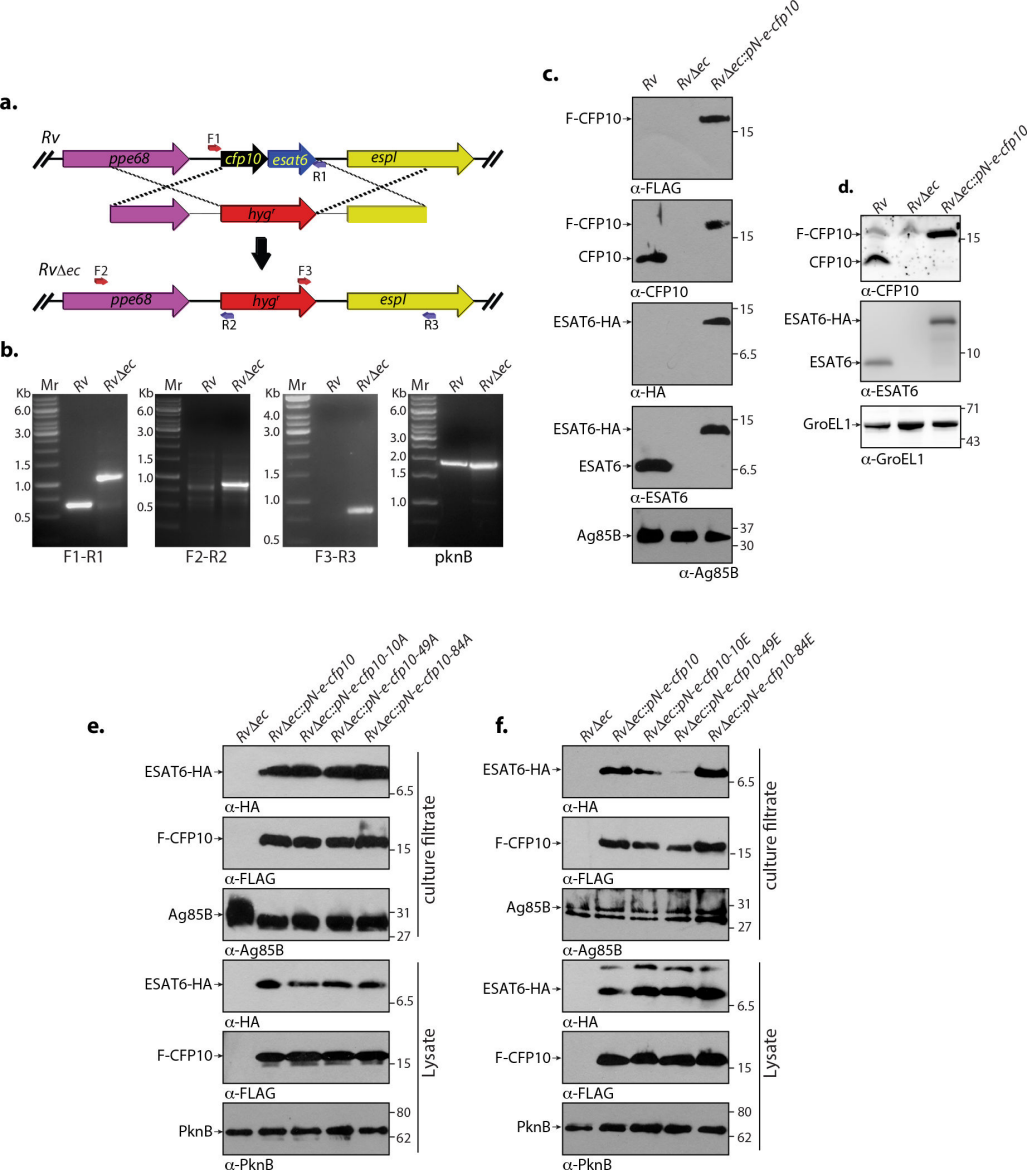
Phosphorylation of CFP10 negatively modulates the secretion of ESAT6. (a) Schematic representation for the generation of *Rv*Δ*ec*. Primers used for PCR confirmation are indicated. (b) Genomic DNA was isolated from logarithmic phase cultures of wild-type and mutant strains, and PCRs were carried out with indicated sets of primers. Mr denotes 1 kb ladder. First panel shows differential amplification of *cfp10-esat6* in *Rv* (0.6 kb) and *Rv*Δ*ec* (1.1 kb). Second and third panels show PCR amplification (0.8 and 0.8 kb, respectively) obtained only in *Rv*Δ*ec*. The last panel shows control *pknB* amplicon (1.8 kb) in both the strains. (c, d) *Rv*Δ*ec* strain was electroporated with pN-e-cfp10 constructs expressing CFP10-ESAT6 in episomal pNit-3F (IVN inducible) vector to generate *Rv*Δ*ec::pN-e-cfp10*. (c) *Rv*, *RvΔec,* and *Rv*Δ*ec::pN-e-cfp10* strains were seeded at an initial *A*_600_ of 0.1 and induced with 5 µM IVN for 3 days. Twenty micrograms of culture filtrates was resolved, transferred to nitrocellulose membrane, and probed with α-HA, α-ESAT6, α-FLAG, α-CFP10, and α-Ag85B antibodies. Endogenous CFP10 or ESAT6 and F-CFP10 or ESAT6-HA are indicated by arrows. (d) *Rv*, *Rv*Δ*ec*, and *Rv*Δ*ec::pN-e-cfp10* strains were seeded at an initial *A*_600_ of 0.1 and induced with 5 µM IVN for 3 days. WCLs were prepared, and 50, 50, and 10 µg were resolved, transferred to nitrocellulose membrane, and probed with α-CFP10, α-ESAT6, and α-GroEL1 antibodies, respectively. Endogenous CFP10 or ESAT6 and F-CFP10 or ESAT6-HA are indicated by arrows. (e, f) *Rv*Δ*ec* strain was electroporated with pN-e-cfp10 or pN-e-cfp10-10A, or pN-e-cfp10-10E, pN-e-cfp10-49A, or pN-e-cfp10-49E or pN-e-cfp10-84A, or pN-e-cfp10-84D constructs expressing CFP10-ESAT6 or CFP10_mutants_-ESAT6 in episomal pNit-3F (IVN inducible) vector to generate *Rv*Δ*ec::pN, Rv*Δ*ec::pN-e-cfp10, Rv*Δ*ec::pN-e-cfp10-10A, Rv*Δ*ec::pN-e-cfp10-10E, Rv*Δ*ec::pN-e-cfp10-49A, Rv*Δ*ec::pN-e-cfp10-49E, Rv*Δ*ec::pN-e-cfp10-84A,* and *Rv*Δ*ec::pN-e-cfp10-84D* strains. (e) Fresh cultures of *Rv*Δ*ec, RvΔec::pN-e-cfp10, Rv*Δ*ec::pN-e-cfp10-10A, Rv*Δ*ec::pN-e-cfp10-49A,* and *Rv*Δ*ec::pN-e-cfp10-84A* strains were seeded at an initial *A*_600_ of 0.1 and induced with 5 µM IVN for 3 days. Culture filtrates and WCLs were prepared. Twenty micrograms of culture filtrates was resolved, transferred to nitrocellulose membrane, and probed with α-HA, α-FLAG, and α-Ag85B antibodies (top panel). 100, 100, and 20 µg of WCLs were resolved and probed with α-HA, α-FLAG, and α-PknB antibodies, respectively (bottom panel). (f) Fresh cultures of *Rv*Δ*ec, Rv*Δ*ec::pN-e-cfp10, Rv*Δ*ec::pN-e-cfp10-10E, Rv*Δ*ec::pN-e-cfp10-49E,* and *Rv*Δ*ec::pN-e-cfp10-84D* strains were seeded at an initial *A*_600_ of 0.1 and induced with 5 µM IVN for 3 days. Culture filtrates and WCLs were prepared. Twenty micrograms of culture filtrates was resolved, transferred to nitrocellulose membrane, and probed with α-HA, α-FLAG, and α-Ag85B antibodies (top panel). 100, 100, and 20 µg of WCLs were resolved and probed with α-HA, α-FLAG, and α-PknB antibodies, respectively (bottom panel). For panels e and f, representative data of two independent experiments are presented.

We subsequently set out to analyze the role of CFP10 phosphorylation on the secretion of either CFP10 or ESAT6. To investigate the role of phosphorylation in the secretion of this dimeric complex, we generated *Mtb*Δ*cfp10esat6* (*Rv*Δ*ec*) mutant strain, wherein both *cfp10-esat6* genes were replaced by *hyg^r^* gene that does not contain any promoter (Fig. 6a). The fidelity of homologous recombination at the native locus was validated by PCR using primers flanking the replacement junctions (Fig. 6b). Analysis of WCL and culture filtrates obtained from *H37Rv*, *Rv*Δ*ec,* and *Rv*Δ*ec::pN-e-cfp10* strains, with α-CFP10 and α-ESAT6 antibodies, revealed (i) the expression levels of F-CFP10 and ESAT-HA were similar to their endogenous expression and (ii) the secretion of CFP10 and ESAT6 is not affected by tags at the N and C-terminus, respectively (Fig. 6c and d).

We analyzed the role of CFP10 phosphorylation on their secretion by transforming *Rv*Δ*ec* with the wild-type, phosphoablative, and phosphomimetic constructs. The protein expression of phosphoablative and phosphomimetic mutants in the whole-cell lysates of *Rv*Δ*ec* transformants was found to be similar to the expression of CFP10 & ESAT6 from *Rv*Δ*ec::pN-e-cfp10* (Fig. 6e and f; bottom panels). The secretion of phosphoablative mutants prepared from *RvΔec* transformants was similar to that of F-CFP10 and ESAT6-HA from *Rv*Δ*ec::pN-e-cfp10* (Fig. 6e). While the F-CFP10 and ESAT6-HA expressions from the phosphomimetic mutants remain unaltered, we observed a partial reduction in the secretion of ESAT6-HA upon phosphorylation of the T10 site of CFP10 (Fig. 6f). Importantly, we observed a marked reduction in the secretion of ESAT6-HA when the T49 site in the CFP10 was mutated to phosphomimetic E residue (Fig. 6f). Together, results suggest that the phosphorylation status of CFP10 at T49 residue regulates secretion of ESAT6.

### Phosphoablative and mimetic mutants of CFP10 show compromised *ex vivo* and *in vivo* survival

**Fig 7:**
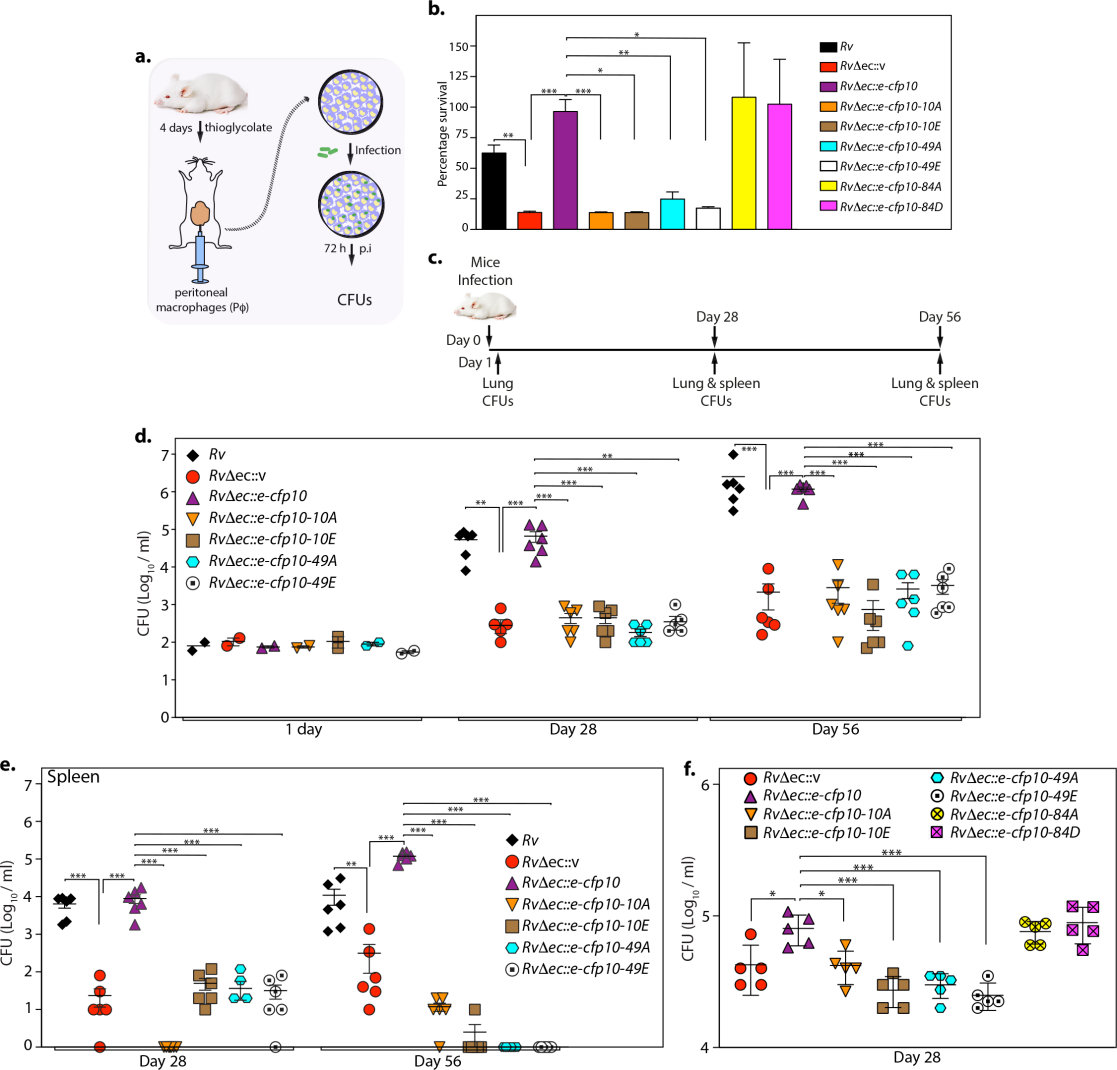
Phosphoablative and mimetic mutants of CFP10 show compromised *ex vivo* and *in vivo* survival. (a) Schematic outline for the peritoneal macrophage infection experiment. (b) *Rv*Δ*ec* strain was electroporated with pST-e-cfp10 or pST-e-cfp10-10A, or pST-e-cfp10-10E, pST-e-cfp10-49A, or pST-e-cfp10-49E or pST-e-cfp10-84A, or pST-e-cfp10-84D constructs expressing CFP10-ESAT6 or CFP10_mutants_-ESAT6 in episomal pST-KT (tet inducible) vector to generate *Rv*Δ*ec::v, RvΔec::e-cfp10, Rv*Δ*ec::e-cfp10-10A, Rv*Δ*ec::e-cfp10-10E, RvΔec::e-cfp10-49A, Rv*Δ*ec::e-cfp10-49E, Rv*Δ*ec::e-cfp10-84A,* and *Rv*Δ*ec::e-cfp10-84D* strains. Above strains were used to infect peritoneal macrophages, at 1:10 m.o.i. CFUs were enumerated 72 h post infection. Error bars represent standard error. Statistical analysis was performed with the help of two-way ANOVA using GraphPad PRISM 6. *P ≤ 0.01, **P ≤ 0.001, ***P ≤ 0.0001. (c) Schematic outline of the murine infection experiment. (d) BALB/c mice were aerosolically infected with 200CFU/mouse of *Rv*, *Rv*Δ*ec::v, Rv*Δ*ec::e-cfp10, Rv*Δ*ec::e-cfp10-10A, Rv*Δ*ec::e-cfp10-10E, Rv*Δ*ec::e-cfp10-49A,* and *Rv*Δ*ec::e-cfp10-49E* strains. CFUs were enumerated in the lungs of infected mice after day 1 (2 mice/group), after 4 weeks (6 mice/group), and 8 weeks (6 mice/group) post infection. After 4 weeks, mean CFU values in the lungs of mice infected with *Rv*, *Rv*Δ*ec::v, Rv*Δ*ec::e-cfp10, Rv*Δ*ec::e-cfp10-10A, Rv*Δ*ec::e-cfp10-10E, Rv*Δ*ec::e-cfp10-49A,* and *Rv*Δ*ec::e-cfp10-49E* were 4.61, 2.03, 4.69, 2.53, 2.53, 2.21, and 2.11 on log10 scale, respectively. After 8 weeks, mean CFU values were 6.14, 2.87, 6.05, 3.05, 2.43, 3.08, and 3.3 on log10 scale, respectively. (e) CFUs were enumerated in the spleen of infected mice after 4 weeks (6 mice/group) and 8 weeks (6 mice/group) post infection. After 4 weeks, CFU values in the spleen of mice infected with *Rv*, *Rv*Δ*ec::v, Rv*Δ*ec::e-cfp10, Rv*Δ*ec::e-cfp10-10A, Rv*Δ*ec::e-cfp10-10E, Rv*Δ*ec::e-cfp10-49A,* and *Rv*Δ*ec::e-cfp10-49E* were 3.72, 1.06, 3.85, 0, 1.55, 1.08, and 1.19 on log10 scale, respectively. After 8 weeks, mean CFU values were 3.75, 1.93, 5.06, 0.93, 0.17, 0, and 0 on log10 scale, respectively. (f) BALB/c mice were aerosolically infected with *Rv* or *RvΔec* strain electroporated with integrative constructs harboring either wild type or mutants of esat6-cfp10. CFUs were enumerated in the lungs of infected mice 28 days post infection. Error bars: S.E.M. Statistical analysis was performed with the help of two-way ANOVA using GraphPad PRISM 6. **P ≤ 0.001, ***P ≤ 0.0001.

Once macrophages phagocytize the pathogen, Esx-1 positive *Mtb* actively inhibits the process of phagosome-lysosome fusion and can escape into the cytosol (63). To elucidate the effect of CFP10 phosphorylation in the survival post-infection, we performed *ex vivo* infection experiments using peritoneal macrophages from BALB/c mice. Peritoneal macrophages were infected with *Rv* or *Rv*Δ*ec::v* or *Rv*Δ*ec::e-cfp10* or *Rv*Δ*ec* complemented with an episomal plasmid expressing phosphoablative or phosphomimetic CFP10 (Fig. 7a). Colony-forming units (CFUs) were enumerated at 72 h post infection to determine the biological impact of the phosphorylation on the intracellular growth and survival of bacteria. The CFUs obtained at 72 h were normalized with respect to CFUs obtained at 0 h for each strain, and percent survivals were plotted (Fig. 7b). As anticipated, the *RvΔec::v* strain exhibited reduced survival (13.9%) compared with the wild-type *Rv* (62.5%) (Fig. 7b). Complementation with wild-type copy restored the compromised phenotype (*Rv*Δ*ec::e-cfp10-*96.4%). However, complementation with both phosphoablative and phosphomimetic mutations of CFP10 (*Rv*Δ*ec::e-cfp10-10A*, *Rv*Δ*ec::e-cfp10-10E, Rv*Δ*ec::e-cfp10-49A,* and *Rv*Δ*ec::e-cfp10-49E*) failed to restore the CFUs (13.7%, 13.7%, 24.9%, and 17.5%, respectively). Phosphoablative or mimetic mutant of S84 residue (*Rv*Δ*ec::e-cfp10-84A* or *Rv*Δ*ec::e-cfp10-84D*) did not impact bacillary survival, suggesting that phosphorylation of S84 is not crucial for its function.

**Fig 8:**
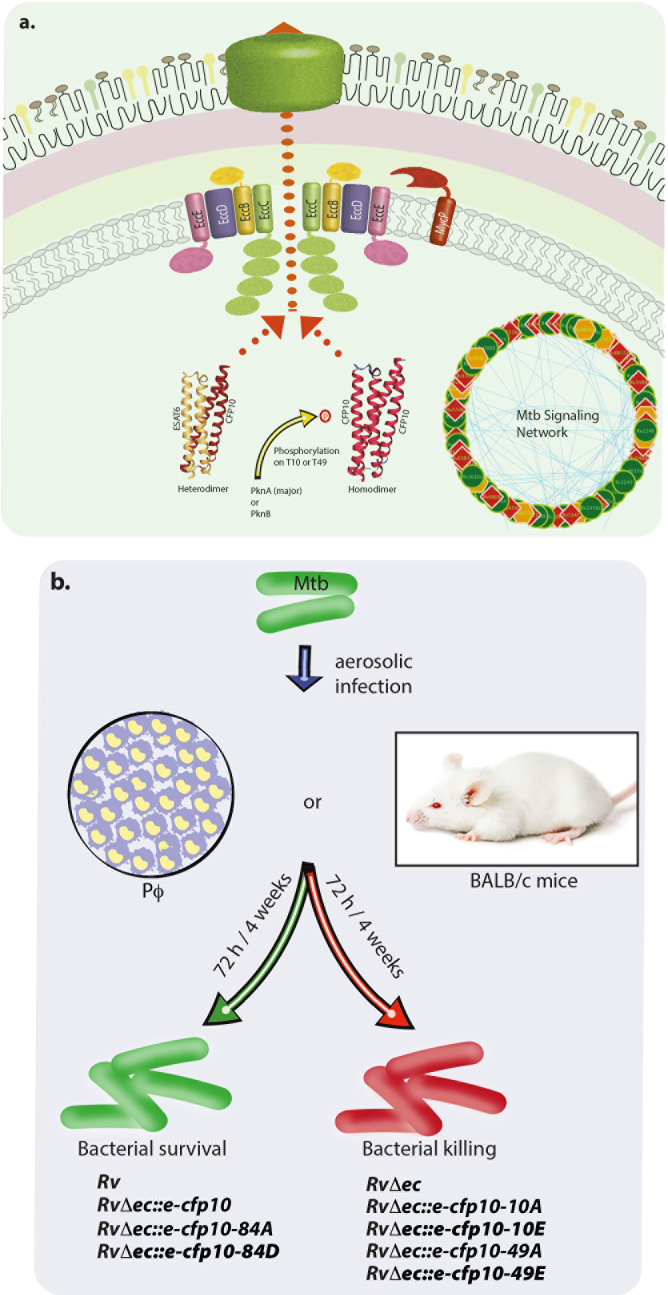
Schematic model. Model is based on the interpretation of our data and previous literature. (a) Based on the signaling network analysis of high-throughput secretome, phosphoproteome, and phospho-secretome, we hypothesized that processes of secretion and phosphorylation are linked. CFP10, which gets phosphorylated on T10 and T49 mostly by PknA and to a limited extent by PknB, was selected to investigate the role of phosphorylation on secretion. While the phosphoablative mutants seem to be efficiently secreting both ESAT6 and CFP10, phosphomimetic mutants showed compromised secretion of ESAT6. We speculate that this could be due to a probable shift toward CFP10 homodimers. (b) *Ex vivo* and murine infection experiments in the peritoneal macrophages with wild type and mutant *Rv, RvΔec::v, Rv*Δ*ec::e-cfp10,* and *RvΔec::e-*cfp10_mut_ revealed that both phosphoablative and phosphomimetic mutants showed compromised survival in the host. These results suggest yet unidentified role for the hydroxyl groups of T10 and T49 in modulating the function of CFP10 in the host.

Next, we validated the importance of CFP10 phosphorylation in the host using the murine infection model (Fig. 7c). BALB/c mice were infected with *Rv*, *Rv*Δ*ec::v*, *Rv*Δ*ec::e-cfp10*, *Rv*Δ*ec::e-cfp10-10A*, *Rv*Δ*ec::e-cfp10-10E, Rv*Δ*ec::e-cfp10-49A,* and *Rv*Δ*ec::e-cfp10-49E* strains through the aerosol route. CFUs were enumerated following one day of infection to check the initial bacterial load, and this was found to be similar for all of the strains (Fig. 7d). After 4 and 8 weeks of infection, the lungs and spleens of mice infected with *Rv* and *RvΔec::e-cfp10* strains showed substantial bacterial survival. On the other hand, lungs and spleen from mice infected with the mutant (*RvΔec::v*) showed significantly reduced survival (Fig. 7d and e). The gross evaluation of infected lungs and spleens (Fig. S5) was following the CFU data. Notably, phosphoablative and phosphomimetic mutations of CFP10 failed to restore the compromised phenotype in both lung and spleen (Fig. 7d and e). The data suggest that the hydroxyl groups play a vital role in the secretion of CFP10. Alternatively, the observed results may be due to the loss of episomal constructs during infection. To address this possibility, we generated complementation strains where cfp10-esat6 were cloned in an integrative construct. The expression of genes from integrative constructs was verified with the help of qRT-PCR (data not shown). We performed mice infection experiments with the new strains (Fig. 7f), and the results obtained were similar to those obtained with episomal construct complementation strains. Thus, the data suggest the engagement of pathogen with the host is a function of the phosphorylation status of CFP10, an exciting new prospect for developing novel therapeutic interventions (Fig. 8).

## DISCUSSION

The development of mass spectrometry techniques has allowed researchers to identify the phosphoproteome of *Mtb*, yet the true extent of phosphorylation remains to be discovered due to technical shortcomings. In a previous study, Prisic et al. reported more than 500 phosphorylation sites in 301 proteins under 6 different culture conditions, each with distinct phosphorylation patterns, in the laboratory strain *H37Rv* (47). In another study, Kusebauch et al. identified 17 proteins with tyrosine phosphorylation (64). Comprehensive analysis of a *Mtb* isolate SAW 5527 identified 214 phosphorylated proteins with 414 phospho-ser/thr/tyr residues, of which 169 proteins were unique (48). In a comparative study between the pathogenic strain *H37Rv* and non-pathogenic strain *H37Ra*, Verma et al. identified 512 phosphosites in 257 proteins, of which 265 sites were novel (49). Recently, in a multisystem analysis of control and inhibitor-treated (against essential kinases, PknA and PknB) strains, 1,241 unique phosphorylation sites were identified in 470 proteins (50). Another study by Zeng et al. identified a total of 712 phosphorylated proteins in *H37Rv* PknA and PknB-depletion strains in the presence or absence of the inducer (57). The current study expanded the phosphoproteome knowledge by identifying 903 phosphorylation sites in 566 *Mtb* proteins.

Analysis of the 4 previous studies with our study revealed that 49 phosphoproteins were common. Notably, we identified 83 phosphoproteins for the first time. The studies mentioned above clearly show that results from single shotgun phosphoproteomics vary significantly from sample to sample. The complexity of phosphoproteome in *Mtb* demands further investigation. This study found 199 proteins common to all 3 biological replicates, suggesting that these proteins are robustly phosphorylated. Moreover, 41 out of the 49 phosphoproteins common to all 5 studies were part of the 199 proteins. The common proteins include FHA-domain containing FhaA, GarA, STPKs-PknA, PknB, cell division proteins—FtsQ, FtsY, and Tat; secretion system protein—TatA; and cell wall synthesis proteins—MviN and GlmM (49, 51, 64 – 68). Among the 11 STPKs, PknA, PknB, PknD, PknF, and PknH were identified in all 3 biological replicates. PknE and PknK were identified in two biological replicates, while PknG was found in one biological replicate. We also identified tyrosine kinase PtkA among the phosphorylated proteins. We have identified 16 tyrosine-phosphorylated proteins, including previously reported, FhaA. FhaA is the most extensive tyrosine-phosphorylated protein in *Mtb*, with nine phospho-tyrosine residues identified to date, and four out of the nine were identified in the current study (48, 64). FhaA is an FHA domain-containing protein and is essential for *in vitro* growth. FhaA-PknB-MviN complex is essential for cell wall synthesis (68). However, the role of tyrosine phosphorylation on FhaA has not yet been elucidated. Together, our study extends the existing knowledge of mycobacterial phosphoproteome. It forms the basis to explore the significance of phosphorylation in different molecular mechanisms. We used high-throughput mass spectrometry to identify the secretome of mycobacteria. To delineate the secretome of *Mtb*, two-dimensional gel electrophoresis and LC-MS/MS were used in previous studies. Malen et al. opted for 2-D gel electrophoresis with LC-MS/MS and identified 257 proteins, of which 159 were most likely secreted by the general secretory pathway (52, 53). De Souza et al. identified 458 proteins in the culture filtrate of *Mtb*, intending to study signal peptide cleavage patterns in secreted proteins (52). After applying several selection criteria, Mehra et al. generated a library of possible secretome ORFs by combining experimental and predicted secreted proteins. This list consists of 339 proteins (54). Analysis of secretory proteins found in any 2 biological replicates revealed that ~27.2% (79 out of 290) of experimentally secreted proteins identified in this study possess a signal peptide, and ~3.1% of proteins (9 out of 290) contain a TAT signal, which is in corroboration with previous studies (Fig. 2). Interestingly, among the 61 proteins found in all four studies, the signal peptide is present in ~63.9% proteins, while the TAT signal is present in ~9.8% proteins, suggesting that these are reliably targeted for secretion. We also performed trans-membrane domain prediction analysis in our experimental secretome and identified that only 5 out of 79 proteins carrying signal peptide have a transmembrane domain, which is as per the general features of all secretory proteins (69) (Fig. 2). Thus, the current study offers a broader picture of the secretome and provides a comparative analysis with the previous studies.

Protein-protein interactions are responsible for the intricate signal transduction pathways in a cell and are critical to understanding the biological system. The network topology was delineated by Wang et al. with the help of high-throughput yeast two-hybrid system, which identified 2,907 proteins (74.1% of functional proteome), forming an extensively connected network through 8,042 protein-protein interactions (56). To better understand the functional organization of the experimental phosphoproteome, secretome, and phospho-secretome, we adopted a computational method to map them onto this PPI network of *Mtb* (56). Subsequent graph-theoretic analysis of the resulting network revealed very interesting *Mtb* PPI network architecture, wherein the phosphorylated (P), secreted (S), and phosphorylated and secreted (PS) proteins formed a substantial network with 3,853 protein-protein interactions. These observations led us to hypothesize if phosphorylation and secretion are related processes in *Mtb*. The most exciting outcome of this analysis is the identification of CFP10 and ESAT6 as critical nodes of the network. Several studies attribute CFP10 and ESAT6 as the major virulence factor of *Mtb*, and lack of their secretion in Δ*esx-1* strain or *ΔPhoP* strain results in compromised virulence (70, 71). Given that it was only pertinent to explore whether phosphorylation by any means impacts their secretion as well. *In vitro* kinase assay showed that CFP10 is majorly phosphorylated by essential kinase PknB and, to some extent, by PknA; *in vivo* studies using both *pknA* and *pknB* conditional mutant strains, we find that PknA is majorly responsible for CFP10 phosphorylation (Fig. 4). This finding corroborates with a previous high-throughput study by Carette et al. where PknA and PknB-inhibitor-treated samples showed decreased phosphorylation of CFP10 (51). Multiple studies have also reported reduced phosphorylation of proteins involved in secretion pathways upon PknA or PknB depletion (51, 72). A recent study where high-throughput phosphoproteomics was performed with PknA and PknB conditional depletion strains also showed that CFP10 is a substrate of PknA (57).

CFP10 and ESAT6 interact with each other in solution along the length of the α-helix (31). The genes encoding these two proteins are co-transcribed and supposedly interact immediately after translation, and the acetylated form of ESAT6 at Thr2 has a somewhat lesser affinity toward CFP10 *in vitro* (73). However, the regulatory impact of other post-translational modifications on CFP10-ESAT6 interactions *in vivo* has not been investigated. While phosphorylation of CFP10 does not seem to influence its interaction with ESAT6, results show that phosphorylation of CFP10 on T49 impacts the secretion of ESAT6 (Fig. 5 and 6). We speculate that in the T49 mutant, CFP10 is secreted as a homodimer instead of a heterodimer. This hypothesis originates from the fact that only homologs of CFP10 exist in firmicutes and are secreted as dimers (74). In fact, in *M. marinum*, mutations in some other non-essential *esx-1* genes, homologous to Rv3876, Rv3878, and Rv3879c in *Mtb,* result in the hampered secretion of ESAT6, but the secretion of CFP10 is not affected (34).

Moreover, using structural approaches, Rosenberg et al. showed that in the absence of CFP10, EccC, the ATPase responsible for the transport of CFP10-ESAT6 complex, exists in a monomeric form duly inactivated by the interaction between ATPase1 and ATPase2 domains (75). It is postulated that CFP10 homodimers are responsible for activating this multimeric complex, whereas the addition of ESAT6 inhibited the ATPase activity and caused cooperative disassembly (75). In *Pseudomonas aeruginosa*, PpkA (a homolog of PknB)-mediated phosphorylation of Fha1 inside cytoplasm leads to the recruitment of type VI secretion system toward the inner membrane that leads to the secretion of periplasm-located Hcp1 across the outer membrane (76). In the same bacteria, type II secretion of proteases is regulated through phosphorylation of flagellin protein, FliC (77). However, this is the first report in *Mtb,* wherein phosphorylation is shown to modulate the secretion of a protein.

Out of five Esx systems, only three, Esx-1, Esx-3, and Esx-5, are shown to secrete substrates (78) actively in *Mtb*. Esx-1 system is involved in various immunomodulatory functions, including escape from phagosome to the cytosol (63, 79 – 81), apoptosis (82, 83), downregulation of IFN- γ (84, 85), suppression of antigen presentation (86), downregulation of autophagy (39, 87), inhibition of ROS production (38), and others. Since CFP10 and ESAT6 are the major secretory proteins of the Esx-1 system, we investigated the effect of their phosphorylation in the virulence of the pathogen. To our surprise, not only the mutant (*RvΔec*) but also both phosphomimetic and ablative mutants (10A and 49A) showed compromised survival in macrophages and mice (Fig. 7). This finding reveals that both unphosphorylated and phosphorylated forms of CFP10 are involved in modulating host functions, which help bacterial survival, underscoring the importance of dynamic protein phosphorylation of CFP10. We were expecting a reduction in virulence with the phosphomimetic CFP10 mutants, but the decreased survival of the phosphoablative mutants was an unexpected finding. Our hypothesis is that the modified phosphorylation state of CFP10 may impact the complex’s stability and that a precise balance between phosphorylated and unphosphorylated forms of CFP10 is crucial for the complex’s subsequent activity.

To conclude, this study establishes that phosphorylation plays an essential role in regulating Esx-1-mediated activity post infection and is crucial for the intracellular survival of the pathogen. The findings that CFP10 phosphorylation regulates bacterial survival during infections also unravel potential new mechanisms for therapeutic intervention. Since Esx-1 is involved in diverse immunomodulatory activities, the possibility of other as yet unknown roles of CFP10-ESAT6 complex that might need regulated phosphorylation seems highly plausible.

## MATERIALS AND METHODS

### Strains, media, and reagents

*M. tuberculosis H37Rv* (ATCC) and *M. smegmatis mc^2^155* (ATCC) were used as wild-type mycobacterial strains. *E. coli DH5α* (Invitrogen) was used for all cloning experiments, while *E. coli* BL21 (DE3) Codon Plus (Stratagene) cells were used for purification of recombinant proteins. *Ms* (*mc^2^155*) and *Mtb* (*H37Rv*) strains were grown in Middlebrook 7H9 media (Difco) supplemented with 10% ADC (albumin, dextrose, and catalase) at 100 rpm at 37°C with appropriate antibiotics when needed. Pristinamycin 1A was procured from Molcon corp, Canada. Isovaleronitrile (IVN) and anhydrotetracycline (ATc) were purchased from Sigma. Restriction endonucleases and other DNA-modifying enzymes were purchased from New England Biolabs. γ[^32^P] ATP was purchased from Perkin-Elmer Life Sciences. Sequencing grade trypsin was obtained from Promega. Hypersep SCX SPE columns were purchased from Thermo Scientific. Phos Select iron affinity gel was purchased from Sigma. Sep-Pak cartridges were obtained from Waters. C18 stage tips were purchased from Nest Group. Both anti-FLAG M2 agarose beads and EZView red anti-HA affinity gel were purchased from Sigma. Constructs used/generated in the study are provided in Table 1. Strains used/generated in the study are provided in Table 2.

**TABLE 1:** Plasmids used in this study

Constructs	Description	Source
pDuet-MBP	*MBP* cloned into NdeI/EcoRV of MCS2 of pDuet	(59)
pDuet-MBP-pknA	*MBP-pknA* cloned into NdeI/EcoRV of MCS2 of pDuet	(59)
pDuet-MBP-pknB	*MBP-pknB* cloned into NdeI/EcoRV of MCS2 of pDuet	(59)
pDuet-MBP-pknD-KD	*MBP-pknD-KD* cloned into NdeI/EcoRV of MCS2 of pDuet	(59)
pDuet-MBP-pknE-KD	*MBP-pknE-KD* cloned into NdeI/EcoRV of MCS2 of pDuet	(59)
pDuet-MBP-pknF-KD	*MBP-pknF-KD* cloned into NdeI/EcoRV of MCS2 of pDuet	(59)
pDuet-MBP-pknG	*MBP-pknG* cloned into NdeI/EcoRV of MCS2 of pDuet	(59)
pDuet-MBP-pknH-KD	*MBP-pknH-KD* cloned into NdeI/EcoRV of MCS2 of pDuet	(59)
pDuet-MBP-pknI-KD	*MBP-pknI-KD* cloned into NdeI/EcoRV of MCS2 of pDuet	(59)
pDuet-MBP-pknJ-KD	*MBP-pknJ-KD* cloned into NdeI/EcoRV of MCS2 of pDuet	(59)
pDuet-MBP-pknK	*MBP-pknK* cloned into NdeI/EcoRV of MCS2 of pDuet	(59)
pDuet-MBP-pknL-KD	*MBP-pknL-KD* cloned into NdeI/EcoRV of MCS2 of pDuet	(59)
pET28b-cfp10	*cfp10* cloned into NdeI-HindIII sites of pET28b	This study
pNit-3F	pNit with 3X-FLAG tag at N-terminus and 3X-HA tag at C-terminus	(72)
pN-F-cfp10	*cfp10* cloned into NdeI-HindIII sites of pNit-3F	This study
pN-e-cfp10	*cfp10-esat6* cloned into NdeI-HindIII sites of pNit-3F	This study
pN-e-cfp10-10A	*cfp10 _T10A_-esat6* cloned into NdeI-HindIII sites of pNit-3F	This study
pN-e-cfp10-10E	*cfp10 _T10E_-esat6* cloned into NdeI-HindIII sites of pNit-3F	This study
pN-e-cfp10-49A	*cfp10 _T49A_-esat6* cloned into NdeI-HindIII sites of pNit-3F	This study
pN-e-cfp10-49E	*cfp10 _T49E_-esat6* cloned into NdeI-HindIII sites of pNit-3F	This study
pN-e-cfp10-84A	*cfp10 _S84A_-esat6* cloned into NdeI-HindIII sites of pNit-3F	This study
pN-e-cfp10-84D	*cfp10 _S84D_-esat6* cloned into NdeI-HindIII sites of pNit-3F	This study
pST-e-cfp10	*cfp10-esat6* cloned into NdeI-HindIII sites of pST-KT	This study
pST-e-cfp10-10A	*cfp10 _T10A_-esat6* cloned into NdeI-HindIII sites pST-KT	This study
pST-e-cfp10-10E	*cfp10 _T10E_-esat6* cloned into NdeI-HindIII sites pST-KT	This study
pST-e-cfp10-49A	*cfp10 _T49A_-esat6* cloned into NdeI-HindIII sites pST-KT	This study
pST-e-cfp10-49E	*cfp10 _T49E_-esat6* cloned into NdeI-HindIII sites pST-KT	This study
pST-e-cfp10-84A	*cfp10 _S84A_-esat6* cloned into NdeI-HindIII sites pST-KT	This study
pST-e-cfp10-84D	*cfp10 _S84D_-esat6* cloned into NdeI-HindIII sites pST-KT	This study

**TABLE 2:** Strains used in this study

Strains	Description	Source
DH5α	*E. coli* strain used for cloning experiments	Invitrogen
BL21 DE3 Codon Plus	*E. coli* strain used for protein expression	Stratagene
*Ms*	Wild-type *M. smegmatis* strain *mc^2^155*	ATCC, 700084
*H37Rv*	Wild-type *M. tuberculosis* strain	ATCC
*Rv::F-cfp10*	H37Rv electroporated with pN-F-cfp10	This study
*Rv*Δ*A::F-cfp10*	Pristinamycin inducible pknA conditional mutant of H37Rv electroporated with pN-F-cfp10	This study
*Rv*Δ*B::F-cfp10*	Pristinamycin inducible pknB conditional mutant of H37Rv electroporated with pN-F-cfp10	This study
*Ms*Δ*esx-1*	*M. smegmatis esx-1* deletion mutant; Hyg^r^	This study
*Ms*Δ*esx-1::pN*	*Ms*Δ*esx-1* electroporated with pNit-3F construct; Hyg^r^, Kan^r^	This study
*Ms*Δ*esx-1::pN-e-cfp10*	*Ms*Δ*esx-1* electroporated with pN-e-cfp10 construct; Hyg^r^, Kan^r^	This study
*Ms*Δ*esx-1::pN-e-cfp10-10A*	*Ms*Δ*esx-1* electroporated with pN-e-cfp10-10A construct; Hyg^r^, Kan^r^	This study
*Ms*Δ*esx-1::pN-e-cfp10-49A*	*Ms*Δ*esx-1* electroporated with pN-e-cfp10-49A construct; Hyg^r^, Kan^r^	This study
*Ms*Δ*esx-1::pN-e-cfp10-84A*	*Ms*Δ*esx-1* electroporated with pN-e-cfp10-84A construct; Hyg^r^, Kan^r^	This study
*Ms*Δ*esx-1::pN-e-cfp10-10E*	*Ms*Δ*esx-1* electroporated with pN-e-cfp10-10E construct; Hyg^r^, Kan^r^	This study
*Ms*Δ*esx-1::pN-e-cfp10-49E*	*Ms*Δ*esx-1* electroporated with pN-e-cfp10-49E construct; Hyg^r^, Kan^r^	This study
*Ms*Δ*esx-1::pN-e-cfp10-84D*	*Ms*Δ*esx-1* electroporated with pN-e-cfp10-84D construct; Hyg^r^, Kan^r^	This study
*H37Rv*	Wild type *M. tuberculosis* strain	ATCC
*Rv*Δ*ec*	*M. tuberculosis cfp10-esat6* deletion mutant; Hyg^r^	This study
*Rv*Δ*ec::pN*	*Rv*Δ*ec* electroporated with pNit-3F construct; Hyg^r^, Kan^r^	This study
*Rv*Δ*ec::pN-e-cfp10*	*Rv*Δ*ec* electroporated with pN-e-cfp10 construct; Hyg^r^, Kan^r^	This study
*Rv*Δ*ec::pN-e-cfp10-10A*	*Rv*Δ*ec* electroporated with pN-e-cfp10-10A construct; Hyg^r^, Kan^r^	This study
*Rv*Δ*ec::pN-e-cfp10-49A*	*Rv*Δ*ec* electroporated with pN-e-cfp10-49A construct; Hyg^r^, Kan^r^	This study
*Rv*Δ*ec::pN-e-cfp10-84A*	*Rv*Δ*ec* electroporated with pN-e-cfp10-84A construct; Hyg^r^, Kan^r^	This study
*Rv*Δ*ec::pN-e-cfp10-10E*	*Rv*Δ*ec* electroporated with pN-e-cfp10-10E construct; Hyg^r^, Kan^r^	This study
*Rv*Δ*ec::pN-e-cfp10-49E*	*Rv*Δ*ec* electroporated with pN-e-cfp10-49E construct; Hyg^r^, Kan^r^	This study
*Rv*Δ*ec::pN-e-cfp10-84D*	*Rv*Δ*ec* electroporated with pN-e-cfp10-84D construct; Hyg^r^, Kan^r^	This study
*Rv*Δ*ec::v*	*Rv*Δ*ec* electroporated with pST-KTconstruct; Hyg^r^, Kan^r^	This study
*Rv*Δ*ec::e-cfp10*	*Rv*Δ*ec* electroporated with pST-e-cfp10 construct; Hyg^r^, Kan^r^	This study
*Rv*Δ*ec::e-cfp10-10A*	*Rv*Δ*ec* electroporated with pST-e-cfp10-10A construct; Hyg^r^, Kan^r^	This study
*Rv*Δ*ec::e-cfp10-49A*	*Rv*Δ*ec* electroporated with pST-e-cfp10-49A construct; Hyg^r^, Kan^r^	This study
*Rv*Δ*ec::e-cfp10-84A*	*Rv*Δ*ec* electroporated with pST-e-cfp10-84A construct; Hyg^r^, Kan^r^	This study
*RvΔec::e-cfp10-10E*	*Rv*Δ*ec* electroporated with pST-e-cfp10-10E construct; Hyg^r^, Kan^r^	This study
*Rv*Δ*ec::e-cfp10-49E*	*Rv*Δ*ec* electroporated with pST-e-cfp10-49E construct; Hyg^r^, Kan^r^	This study
*Rv*Δ*ec::e-cfp10-84D*	*Rv*Δ*ec* electroporated with pST-e-cfp10-84D construct; Hyg^r^, Kan^r^	This study

### Protein sample preparation for mass spectrometry

For phosphoproteomics and culture filtrate preparation, the strains were first revived in 7H9 media and then grown in Sauton’s media in the presence of 0.005% Tween 80 (Sigma) until the A_600_ reached 0.8–1.0. WCLs were prepared from the cell pellet using the lysis buffer (8 M urea in 25 mM NH_4_HCO_3_) supplemented with appropriate protease and phosphatase inhibitors (Roche). The protein content was estimated using BCA kit (Pierce). Trypsinization and strong cation exchange chromatography (SCX) of 7 mg WCL was performed as described previously (88). In brief, trypsin was added to protein samples at 1:50 ratio (trypsin:protein) after reduction and alkylation and overnight digestion was carried out. Fractions of tryptic digests were collected using bench top SCX cartridges. For that, desalted peptides were resuspended in 2 mL SCX buffer A (7 mM KH_2_PO_4_, pH 2.65, 30% acetonitrile) and loaded onto the column. Salt step elutions were performed in succession, and each fraction was collected (0, 40, 60, 100, and 350 mM KCl in SCX buffer A) followed by desalting of individual fractions using Sep-Pak cartridges. For enrichment of phosphopeptides, peptides from each fraction were resuspended in 1 mL 50% acetonitrile containing 0.1% trifluoroacetic acid and were incubated with 20 µL of metal-based IMAC beads (Phos-Select Iron Affinity Gel) for 60 min on a nutator. The slurry was then transferred to washed and equilibrated C18-stage tips. Phosphopeptides were then eluted from the IMAC resin with three changes of 70 µL IMAC Elution buffer (500 mM K_2_HPO_4_, pH 7.0). At this point, the aqueous-eluted peptides will be bound by the C18 in stage tip. The STAGE-tip was now washed with 50 µL 1% formic acid. Final elution was carried out in 40 µL 70% acetonitrile/0.1% formic acid (89). Enriched fractions were resuspended in 20 µL 5% acetonitrile containing 0.1% formic acid. Two microliters of each fraction was injected into the mass spectrometer (Thermo LTQ Orbitrap Velos).

For culture filtrate preparation, cultures were grown in Sauton’s media as described above, and the culture supernatant was filtered through a 0.2-μm syringe filter to remove the remaining bacteria. CFPs were concentrated using 3 kDa cutoff centricon (Amicon Ultra from Millipore). Protein content was estimated using the BCA kit. Samples were stored at −80°C until further analysis. For proteomic analysis of CFP, 50 µg protein was incubated with urea at a final concentration of 2 M at 37°C for 2 h for denaturation. Proteins were reduced and alkylated as described previously (90), and the urea concentration was reduced to 1 M by adding 25 mM NH_4_HCO_3_. Trypsin was added at 1:50 ratio (trypsin:protein), and digestions were performed at 37°C overnight. Samples were desalted using C18 Stage tips, dried by speed vac, and reconstituted in 50 µL 5% acetonitrile containing 0.1% formic acid. One microliter of sample was injected for MS analysis. For phosphoproteomic analysis of CFP proteins, 2 mg of protein was reduced, alkylated, and digested as described earlier (89). Peptides were desalted using 100 mg Sep-Pak cartridge, eluted in 1.2 mL 80% acetonitrile containing 0.1% trifluoroacetic acid, and dried using speed vac. Phosphopeptides were enriched as described above. Enriched peptides were resuspended in 20 µL 5% acetonitrile containing 0.1% formic acid, and 2 µL was injected for MS analysis.

All proteomics samples were analyzed using the EASY-nLC system (Thermo Fisher Scientific) coupled to LTQ Orbitrap-Velos mass spectrometer (Thermo Fisher Scientific) equipped with a nanoelectrospray ion source. A 10-cm PicoFrit Self-Pack microcapillary column (New Objective) was used to resolve the peptide mixture, and the peptides were eluted as described previously (91). For phosphoproteomic analysis of WCL, a 15-cm column was used. The LTQ Orbitrap-velos was operated using the Top20 CID (High/High) data-dependent acquisition mode with a full scan in the Orbitrap and an MS/MS scan in the CID. The target values for the full scan MS spectra were set at 0.5 × 10^6^ charges with a maximum injection time of 300 ms and a resolution of 60,000 at *m*/*z* 400. Spectra were queried against the *Mtb H37Rv* UniprotKB database. The precursor and fragment mass tolerances were set at 10 ppm and 0.8 Da, respectively. The enzyme specificity was set for Trypsin along with a maximum missed cleavage value of two. Proteome Discoverer 1.3 was used with Sequest as the search algorithm with the oxidation of methionine and carbamidomethylation of cysteine as fixed modification and phosphorylation of serine, threonine, and tyrosine were used as variable modifications. All PSMs were identified at a 1% false discovery rate. For peptide identification, a peptide posterior error probability threshold of 0.01 was specified. Default settings were applied for all other parameters. The probability of phosphorylation at each residue was calculated using the PhosphoRS node in Proteome Discoverer. Phosphopeptides with ≥80% localization probability and pRS score ≥50 were considered for further analysis. The raw MS files mapped the peptide sequence with the Uniprot ID of the respective *Mtb* proteins. A comprehensive and non-redundant list of all *Mtb* proteins along with their Uniprot ID and functional classification was generated using the Mycobrowser database. The Uniprot ID of identified proteins was mapped to their respective gene or Rv number using in-house scripts for further analysis. The mass spectrometry proteomics data have been deposited to the ProteomeXchange Consortium (92) via the PRIDE partner repository with the data set identifier PXD017004.

### Western blot analysis

For western blotting, 10–100 μg of WCL or CFP was resolved on SDS-PAGE followed by transferring to a nitrocellulose membrane. The membrane was then probed with appropriate dilutions of α-FLAG (1:3,000), α-HA (1:4,000), α-Ag85B (1:3,000), α-CFP10 (1:1,000), α-ESAT6 (1:1,000), α-PknA (1:10,000), α-PknB (1:10,000), α-GroEL1 (1:10,000), α-MBP (1:3,000), or α-p-thr (1:1,000) antibodies. Incubation with primary antibody was followed by incubation with HRP-linked anti-mouse/rabbit secondary antibody (Cell Signalling Technology). α-FLAG monoclonal antibody was purchased from Sigma. α-HA monoclonal antibody and α-*p*-threonine antibody were purchased from Cell Signalling Technology. α-Ag85B, α-CFP10 polyclonal antibody, and α-ESAT6 monoclonal antibody were purchased from AbCam. α-MBP antibody was purchased from New England Biolabs. α-PknA, α-PknB, and α-GroEL1 antibodies were raised in rabbit in the lab.

### *In vitro* kinase assay and essential kinase depletion

pQE2-cfp10 construct expressing CFP10 was transformed into *E. coli* BL21 (DE3) Codon Plus cells, and the His-tagged protein was purified as described earlier (59). pDuet-STPK described earlier (59) were transformed into *E. coli* BL21 cells followed by preparation of crude lysate. *In vitro* kinase assay was performed as described previously using the crude lysates and purified substrate, His-CFP10 (59). After the reaction, His-CFP10 was pulled down using Ni-NTA beads (Qiagen) and run on a 16% tris-tricine gel containing 6 M urea.

*Rv, Rv*Δ*B* (*Rv-pptr-B*), and *Rv*Δ*A* (*Rv-pptr-AB::B*) strains were electroporated with pNit-3F-cfp10 construct. Transformants were grown in 7H9 media containing pristinamycin 1A (100 ng/mL) till *A*_600_ reached ~0.8. Cultures were then washed three times with equal volumes of PBST_80_ (1× PBS with 0.05% Tween 80) and diluted to *A*_600_ of ~0.1. *Rv*Δ*B::F-cfp10* was grown for 3 days in the presence or absence of pristinamycin to deplete the kinase PknB. *Rv*Δ*A::F-cfp10* was grown for 3 days in the presence of pristinamycin (100 ng/mL) or in the absence of pristinamycin but with 1.5 µg/mL ATc so that PknB continues to express leading to depletion of only PknA. In all cases, 5 µM IVN was added to express F-CFP10. WCLs were prepared and probed with α-PknB, α-PknA, and α-FLAG antibodies to check for kinase depletion and F-CFP10 expression. F-CFP10 was immunoprecipitated using FLAG-M2 beads and probed with α-p-Thr antibody to check for phosphorylation.

### Generation of gene replacement mutants

*Esx-1* region in *Ms* comprises 27 genes spanning from *sm*_0056 to *sm*_0082. To generate targeted gene disruptions, 1 kb each of 5′ and 3′ flanking regions was PCR amplified using specific primers from *mc^2^155* genomic DNA. PCR amplicons were digested with PflMI and ligated with hyg^r^-sacB and oriE + cos*λ* fragments from pYUB1474 (93) to generate allelic exchange substrate (AES). To generate *Ms*Δ*esx-1*, AES was linearized using a unique EcoRV site, and the linearized substrate was electroporated into pJV53 carrying *Ms* strain induced for recombinase overexpression (94). The *hyg^r^* colonies obtained were screened by PCR, and western blot analysis was performed to confirm recombination at the native locus.

5′ and 3′ flank sequences of *cfp10-esat6* in *Mtb* (~800 bp) were amplified using primers containing DraIII sites on either side. The flanks were digested with DraIII and ligated with PflMI digested oriE + cosλ fragment from pYUB1474, and the PflMI fragment of the PCR amplicon for *hyg^r^* gene without promoter and compatible PflMI digested apramycin resistance gene. The AES was then linearized using SnaBI and electroporated into recombination-proficient strain of *Mtb* carrying pNit-ET plasmid (94). *Rv*Δ*ec* recombinants obtained were screened by PCR amplification using specific sets of primers to ensure proper recombination at the native locus.

### Immunoprecipitation and preparation of CFP

Point mutants were generated with the help of an overlapping PCR method. PCR amplicons of *cfp10-esat6* or *cfp10_mut_-esat6* were cloned into pNit-3F vector (72). In these constructs, CFP10 and CFP10_mut_ proteins would have an N-terminal 3×-FLAG tag, and ESAT6 contains a C-terminal 3×-HA tag. pNit-e-cfp10 and pNit-e-cfp10_mut_ mutant constructs were electroporated into *Ms*Δ*esx-1* strain. The cultures were grown in 7H9 media till *A*_600_ reached ~0.8, and the fresh cultures were induced with 5 µM IVN at an initial *A*_600_ of 0.1. The induced cultures were grown for 10 h, and the WCLs were prepared as described earlier (59). FLAG-tagged CFP10 was immunoprecipitated using FLAG-M2 beads, and HA-tagged ESAT6 was immunoprecipitated using HA EZ-view beads. Immunoprecipitated samples were resolved in a 16% Tris-tricine gel containing 6 M urea followed by western blot analysis.

pNit-3F or pNit-e-cfp10 and pNit-e-cfp10_mut_ constructs were electroporated in *RvΔec* strain to generate *Rv*Δ*ec::pN, Rv*Δ*ec::pN-e-cfp10,* and *Rv*Δ*ec::pN-e-cfp10_mut_* strains. Lysates and CFPs were prepared from cultures induced with 5 µM IVN (3 days). Lysates and CFP were resolved in 16% tris tricine gel containing 6 M urea and analyzed with specific antibodies by western blotting.

### Infection experiment

Peritoneal macrophages were isolated from BALB/c mice 72 h after injecting a 4% thioglycollate solution (Hi-Media). Cells were cultured in RPMI 1640 (Gibco) medium containing 10% heat-inactivated fetal bovine serum (Gibco) and maintained at 37°C. After 12 h, cells were washed with RPMI medium and infected with *Mtb* wild type or mutant or complemented strains. PCR amplicons of *cfp10-esat6 and cfp10_mut_-esat6* were cloned into pST-KT vector (95) to generate pST-e-cfp10 and pST-e-cfp10_mut_ constructs. Vector (pST-KT) or pST-e-cfp10 or pST-e-cfp10_mut_ constructs were electroporated into *Rv*Δ*ec* strain to generate *Rv*Δ*ec::v*, *Rv*Δ*ec::e-cfp10*, *Rv*Δ*ec::e-cfp10-10A*, *Rv*Δ*ec::e-cfp10-10E*, *Rv*Δ*ec::e-cfp10-49A*, *Rv*Δ*ec::e-cfp10-49E*, *Rv*Δ*ec::e-cfp10-84A,* and *Rv*Δ*ec::e-cfp10-84D* strains. Single-cell suspensions (by passing them through a 26-gauze needle 10 times) were prepared from cultures induced with 2 µM ATc. The number of bacteria was quantified by taking the *A*_600_ of the single-cell suspensions, and an appropriate number of bacteria was used for infecting peritoneal macrophages. The bacillary load was determined 72 h post infection.

### Mice infection experiment

Single-cell suspensions of mycobacterial strains *Rv*, *Rv*Δ*ec::v*, *Rv*Δ*ec::e-cfp10*, *Rv*Δ*ec::e-cfp10-10A*, *Rv*Δ*ec::e-cfp10-10E*, *Rv*Δ*ec::e-cfp10-49A,* and *Rv*Δ*ec::e-cfp10-49E* strains were prepared as described above. BALB/c mice of either sex (4–6 weeks old) were obtained from the small animal facility at the National Institute of Immunology and housed in individually ventilated cages at TACF, ICGEB, New Delhi, India. Mice (*n* = 6) were infected with ~200 colony-forming units of each strain by aerosol route as described previously (96). The bacillary load in the lungs was determined 24 h post infection to confirm the implantation. Bacterial loads were determined from the lung and spleen, 4 and 8 weeks post infection to determine the extent of infection and pathogen survival.

### Bioinformatic analysis of secretory proteins

Secreted proteins reported by each experimental study were assessed with bioinformatics-based predictions on secretion signals. Prediction for signal peptide in a protein was made based on two tools, SignalP 6 (97) and Phobius (98), and we report for each protein a combined prediction for the presence of signal peptide using OR rule (SignalP or Phobius). Prediction for TAT signal in a protein was made based on three tools, PRED-TAT (99), Tatfind 1.4 (100), and SignalP 6 (101), and we report for each protein a combined prediction for the presence of TAT signal using majority rule. Prediction of lipoproteins was made based on two tools, PRED-LIPO (102) and SignalP 6, and we report a combined prediction for lipoproteins using OR rule (PRED-LIPO or SignalP). Furthermore, we also assessed the number of proteins reported in each experimental secretome with a predicted transmembrane domain or predicted signal peptide with no transmembrane domain. Phobius and TMHMM 2.0 (103) were used for transmembrane domain predictions, and a combined prediction for the transmembrane domain was made using OR rule (Phobius or TMHMM). The AAR value of proteins in each experimental secretome and complete proteome of *Mtb* was calculated using the predicted number of antigenic regions per protein. The number of antigenic regions per protein in each of the experimental secretome was predicted using BepiPred 2.0 (104) with the default threshold of 0.5 and Kolaskar-Tongaonkar method (105) using EMBOSS antigenic program (106) with a threshold of 1.0. Only predicted antigenic regions with a length ≥6 amino acids were accounted for AAR computation.

### Quantification and statistical analysis

Statistical analysis was performed with the help of two-way ANOVA in GraphPad PRISM 6, and statistical significance is mentioned in the figures and figure legends. In figures, asterisks denote statistical significance (*P ≤ 0.01, **P ≤ 0.001, ***P ≤ 0.0001).

## Data Availability

The mass spectrometry proteomics data have been deposited to the ProteomeXchange Consortium via the PRIDE partner repository with the data set identifier PXD017004. Secretome data have been uploaded in PRIDE under accession number PXD045586.

## SUPPLEMENTAL MATERIAL

Data Set S1 mbio.01232-23-s0001.xlsx Unique phosphorylated proteins and functional categorization.

Data Set S2 mbio.01232-23-s0002.xlsx Unique phosphopeptides.

Data Set S3 mbio.01232-23-s0003.xlsx Phosphorylated proteins and phospho sites.

Data Set S4 mbio.01232-23-s0004.xlsx Secretory proteins common to replicates and total list of secretory proteins.

Data Set S5 mbio.01232-23-s0005.xlsx Secretory proteins in multiple studies.

Data Set S6 mbio.01232-23-s0006.xlsx Attributes of phosphorylated and secreted proteins for the PPI network.

Data Set S7 mbio.01232-23-s0007.xlsx Phosphorylated proteins identified in culture filtrate.

Data Set S8 mbio.01232-23-s0008.xlsx Phospho sites identified in the culture filtrate.

Supplemental Figures mbio.01232-23-s0009.pdf Figures S1 and S2 and legends for Data Sets S1 to S8.

ASM does not own the copyrights to Supplemental Material that may be linked to, or accessed through, an article. The authors have granted ASM a non-exclusive, world-wide license to publish the Supplemental Material files. Please contact the corresponding author directly for reuse.
