# Supplementary material for: Phosphorylation of CFP10 modulates Mycobacterium tuberculosis virulence
Source: mBio. 2023 Oct 4;14(5):e01232-23. doi: 10.1128/mbio.01232-23 (PMC10653824; doi:10.1128/mbio.01232-23)
Supplement: Supplemental Figures — Figures S1 and S2 and legends for Data Sets S1 to S8. [file mbio.01232-23-s0009.pdf]

Supplementary Figures

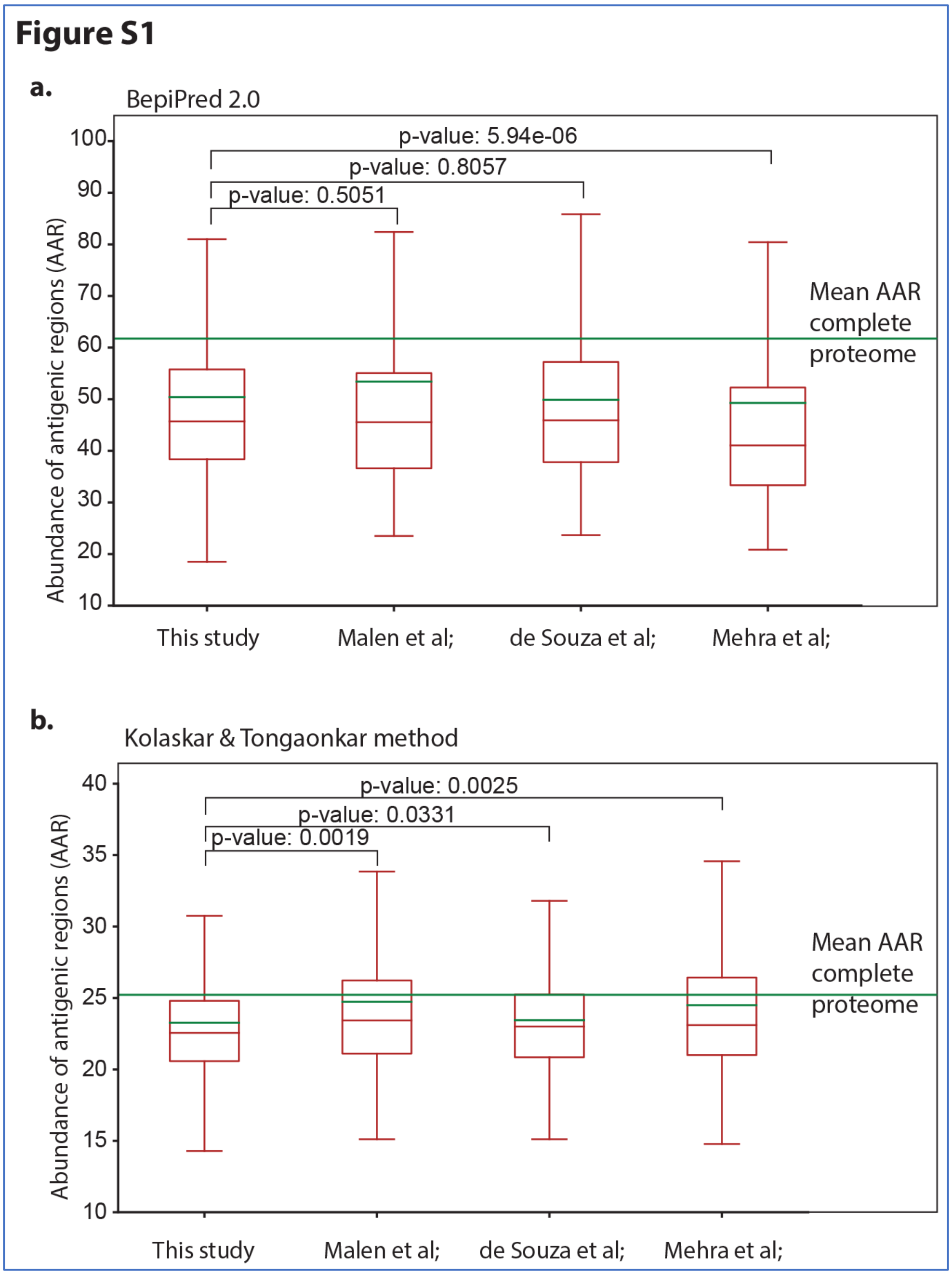

Figures S1. The box plot of computed AAR values for each experimental secretome using (a) BepiPred 2.0 and (b) Kolaskar-Tongaonkar method. In each box plot, the lower end of the box represents the first quartile, brown line inside the box is the median, green line inside the box is the mean and the upper end of the box represents the third quartile of the distribution. Also, the average AAR value of the complete proteome is shown as a horizontal green line in each plot. In this figure, we also report the p-value from the comparison of the distributions of AAR values of experimental secretome from this study along with three other experimental secretomes performed using Wilcoxon rank-sum test.

Figure S2

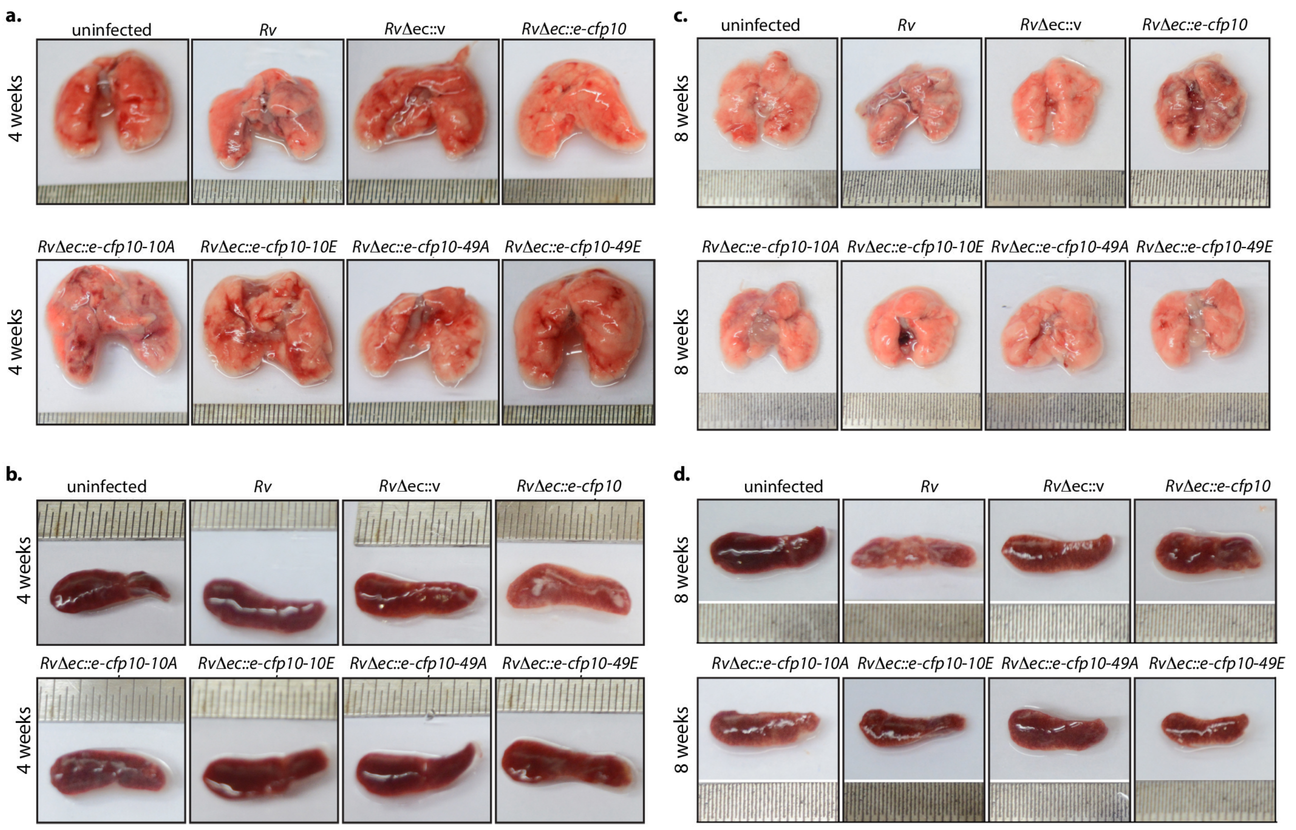

Figure S2. **a & c.** Gross lungs pathology of uninfected, *H37Rv*, *Δce::kv*, *Δce::kce*, *Δce::kce-10A*, *Δce::kce-10E*, *Δce::kce-49A* and *Δce::kce-49E* infected mice 4 (**a**) and 8 weeks (**c**) post infection. **b & d.** Gross spleen pathology of uninfected, *H37Rv*, *Δce::kv*, *Δce::kce*, *Δce::kce-10A*, *Δce::kce-10E*, *Δce::kce-49A* and *Δce::kce-49E* infected mice 4 (b) and 8 weeks post infection.

Supplementary Datasets

Dataset S1a. Unique phosphorylated proteins identified in whole cell lysate (WCL) of *Mtb H37Rv.*

Dataset S1b. Presence of unique phosphorylated protein in different biological replicates

Dataset S2: Unique phosphopeptides identified in *Mtb H37Rv* whole cell lysate (WCL)

Dataset S3a. List of phosphorylated proteins identified in this study and four previous studies.

Dataset S3b. List of phosphosites identified in this study and four previous studies.

Dataset S4a. List of identified secretory proteins in the culture filtrate of *Mtb H37Rv.*

Dataset S4b. Presence of unique proteins in different biological replicates

Dataset S5: List of secretory proteins identified in this study and three previous studies

Dataset S6: Attributes of phosphorylated and secreted proteins for the PPI network

Dataset S7: List of phosphorylated proteins identified in culture filtrate of *Mtb H37Rv*

Dataset S8: List of phospho sites identified in the culture filtrate of *Mtb H37Rv*
